# Directional Three-Dimensional Macroporous Carbon Foams Decorated with WC_1−x_ Nanoparticles Derived from Salting-Out Protein Assemblies for Highly Effective Electromagnetic Absorption

**DOI:** 10.1007/s40820-025-01920-z

**Published:** 2025-09-15

**Authors:** Yongzheng Chen, Lixue Gai, Bo Hu, Yan Wang, Yanyi Chen, Xijiang Han, Ping Xu, Yunchen Du

**Affiliations:** State Key Laboratory of Space Power-Sources, School of Chemistry and Chemical Engineering, Harbin Institute of Technology, Harbin, 150001 People’s Republic of China

**Keywords:** 3D macroporous carbon-based foams, Directional pore channels, Salting-out protein assemblies, EM wave absorption, Directional dependence

## Abstract

Directional three-dimensional carbon-based foams are emerging as highly attractive candidates for promising electromagnetic wave absorbing materials (EWAMs) thanks to their unique architecture, but their construction usually involves complex procedures and extremely depends on unidirectional freezing technique. Herein, we propose a groundbreaking approach that leverages the assemblies of salting-out protein induced by ammonium metatungstate (AM) as the precursor, and then acquire directional three-dimensional carbon-based foams through simple pyrolysis. The electrostatic interaction between AM and protein ensures well dispersion of WC_1−x_ nanoparticles on carbon frameworks. The content of WC_1−x_ nanoparticles can be rationally regulated by AM dosage, and it also affects the electromagnetic (EM) properties of final carbon-based foams. The optimized foam exhibits exceptional EM absorption performance, achieving a remarkable minimum reflection loss of − 72.0 dB and an effective absorption bandwidth of 6.3 GHz when EM wave propagates parallel to the directional pores. Such performance benefits from the synergistic effects of macroporous architecture and compositional design. Although there is a directional dependence of EM absorption, radar stealth simulation demonstrates that these foams can still promise considerable reduction in radar cross section with the change of incident angle. Moreover, COMSOL simulation further identifies their good performance in preventing EM interference among different electronic components.

**Supplementary Information:**

The online version contains supplementary material available at 10.1007/s40820-025-01920-z.

## Highlights

- A groundbreaking approach is developed for the fabrication of directional macroporous WC_1−x_/C foams, which frees the dependence on unidirectional freezing technique from the construction of directional macroporous carbon-based composites.
- The electrostatic interaction between ammonium metatungstate and protein makes in situ generated tungsten carbide (WC_1−x_) nanoparticles well disperse on carbon flakes.
- The optimized foam exhibits exceptional electromagnetic absorption performance, achieving a remarkable minimum reflection loss of − 72.0 dB and an effective absorption bandwidth of 6.3 GHz.

**Supplementary Information:**

The online version contains supplementary material available at 10.1007/s40820-025-01920-z.

## Graphical abstract

**Figure:**
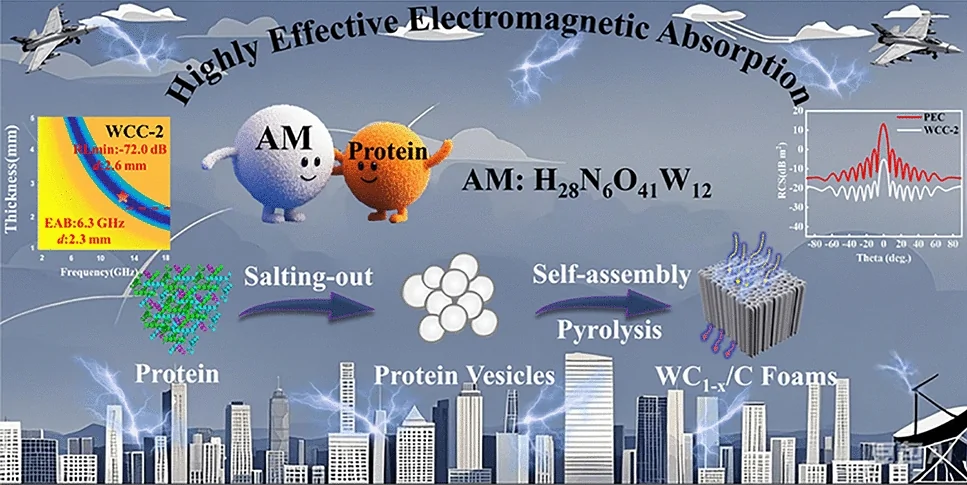

## Introduction

The development of current society, especially in the fields of intelligence and communication, heavily relies on electromagnetic (EM) technology, which inevitably generates excessive EM radiation in human’s living space, leading to a serious situation about EM pollution [1]. Relevant studies indicate that the progressive deterioration of EM environment not only adversely affects the operation of precision equipment but also poses serious health hazards. Consequently, the mitigation of EM pollution is evolving as a critical issue that demands urgent attention worldwide [2–4]. Compared with traditional EM protection based on strong reflection of EM wave, absorption-dominant strategy is gaining more and more concerns, because it provides an effective way to terminate the propagation of EM wave and convert EM energy into other forms [5]. In this context, the research on high-performance EM wave absorbing materials (EWAMs) has become one of the most important topics in the field of materials science. Previous studies have demonstrated that many substances with magnetic or dielectric characteristics, such as magnetic metals and ferrites, carbon materials, carbides, metal oxides/sulfides, conductive polymers, could attenuate incident EM wave to some extent [6–10]. Among all kinds of candidates, carbon materials always reside at the forefront benefited from their low cost, abundant reserve, and tailorable dielectric property [11, 12], and to further strengthen EM absorption performance, carbon-based composites with various secondary EM components are being extensively designed and constructed to improve impedance matching and enrich EM loss mechanisms [13, 14]. Literature review shows that carbides are a class of popular secondary EM components to couple with carbon matrix in recent years, and especially, the emergence of MXenes remarkably stimulates the research interest on carbides/carbon composites as promising EWAMs [15]. However, the easy oxidation of MXenes sets considerable obstacles for the applications of related composites, and thus covalent carbides (e.g., SiC) and interstitial carbides (e.g., ZrC and TiC) usually with strong corrosion resistance, excellent hardness, mechanical strength, and ultra-high melting point are usually regarded as reliable additives in carbon-based composites [16]. Of note is that the formation of carbides is usually achieved at very high temperature (e.g., over 1400 °C for SiC) [17], and thus the products may have large particle size, which inevitably reduces their compatibility in carbon matrix and suppresses the synergistic effects between carbides and carbon matrix [18]. By comparison, tungsten carbide, one of the most important metal carbides with Pt-like catalytic properties, can be easily harvested in carbon matrix under moderate conditions (700–900 °C), and thus they may have much smaller particle size than other carbides [19]. These favorable features actually suggest the potential application in the field of EM absorption. On one hand, the relatively low synthesis temperature makes it easier to tailor the relative graphitization degree of carbon matrix, avoiding the mismatched characteristic impedance, and on the other hand, the small size and uniform dispersion of tungsten carbide nanoparticles can create abundant heterogeneous interfaces, providing powerful interfacial polarization loss [12]. Duan et al. prepared WC/C microspheres by pyrolyzing the mixture of polydopamine and Na_2_WO_4_, and they found that the resultant composite could display both effective EM absorption and good corrosion protection [20]. Lian et al. also fabricated carbon nanosheets decorated by ultrafine WC_1−x_ nanoparticles via a simple solvent-free strategy, and the size of WC_1−x_ nanoparticles was as small as 3–4 nm [21]. The strong synergistic effect between WC_1−x_ nanoparticles and carbon nanosheets endowed the final composite with considerable EM absorption performance, whose strongest reflection loss (RL) and effective absorption bandwidth (EAB) were − 26.3 dB and 4.9 GHz with the absorber thickness of 1.50 mm. Although tungsten carbide/carbon composites have demonstrated their potential as promising EWAMs, there is still plenty of room to upgrade their performance through rational design and optimization.

It should be pointed out that chemical composition is not the only factor to affect the EM properties of carbon-based composites, and microstructure is also extremely important for their EM absorption performance [16, 22]. For example, Tao et al. manipulated the microstructure evolution from solid carbon microspheres to multi-shell hollow porous carbon microspheres, and they found that multi-shell hollow structure could enhance the conductivity loss and polarization loss effectively, thus broadening EAB from 2.3 to 5.2 GHz with a mere thickness of 1.6 mm [23]. In recent research about EM absorption performance of SiC/C composites, Gai et al. further clarified that hollow structure could improve impedance matching under the same volume fraction in resin matrix due to its high porosity, but enhance overall dielectric loss capability under the same mass fraction in resin matrix due to its high specific volume [16]. Apart from those common porous, hollow, and yolk-shell configurations, the construction of 3D macroporous microstructure is emerging as a mainstream design concept in carbon-based EWAMs, because the ultra-large pore size (over tens of microns) therein is relatively close to the wavelength of EM wave, which offers immense potential to induce multiple reflection/scattering of EM wave and thus favors the intensified consumption of EM energy [24, 25]. For example, Zhao et al. assembled carbon nanocoils, graphene, carbon nanotubes, and Fe_2_O_3_ nanoparticles into hierarchical carbon-based aerogel under hydrothermal condition, and the results confirmed that the formation of macroporous structure could pull down the minimum reflection loss (RL) from − 9.1 to − 71.5 dB and EAB was also broadened from 0 to 5.6 GHz [26]. After comprehensive analysis and discussion, they concluded that in addition to the intrinsic loss from different components, hierarchical macroporous structure indeed played an important role in boosting multiple scattering of incident EM waves. More recently, some studies further report the anisotropic dependence of EM absorption performance on the alignment of macroporous structure, and especially when EM waves propagate parallel to the anisotropic channels, there will be much better performance because the open macroporous channels are more favorable for the improvement of impedance matching and loss capacity [27, 28]. In view of these findings, the construction of 3D macroporous carbon-based EWAMs with anisotropic channels is receiving more and more attention in this field [29–31]. For instance, Liang et al. employed directional freezing technology to fabricate a magnetic MXene/graphene aerogel with aligned channels, and they revealed that the oriented pore structure substantially enhanced EM wave dissipation efficiency, achieving a minimum reflection loss of -75.0 dB [28]. Similarly, Chen et al. utilized the same method to prepare carbon nanofibrous aerogels using chitosan as the raw materials. They demonstrated that anisotropic channel alignment not only improved EM attenuation capability but also exhibited remarkable efficacy in optimizing impedance matching [30]. These results inspire us that if we introduced anisotropic 3D macroporous into tungsten carbide/carbon composites, there would be a great opportunity to enhance their EM absorption performance significantly, but the related research is still inaccessible. Recent progress reveals that to date, the only effective way to fabricate anisotropic 3D macroporous carbon-based EWAMs is unidirectional freezing technique, which benefits from the growth of ice crystals along one side of the suspension induced by a steep cooling gradient, thus creating a uniaxial aligned array [32]. It is unfortunate that such a unique technique always needs special customizable equipment and faces a huge challenge in large-scale production. Moreover, unidirectional freezing process mandatorily requires the homogeneous dispersion of different precursors to avoid gradient settlement, and even in that case, the agglomeration of secondary EM components usually occurs in final carbon-based composites [30]. Therefore, it is also extremely in demand to find a simple method for the construction of 3D macroporous tungsten carbide/carbon composites with anisotropic channels.

The conversion of natural plants, such as chitosan, pericarps, wood, is widely accepted as a simple and effective strategy to prepare 3D macroporous carbon materials, because the natural channels from biological tissues can be well preserved after high-temperature pyrolysis [33, 34]. However, the complexity of biological tissues, as well as the obvious differences caused by individuals, regions, and species, makes it rather difficult to ensure the reproducibility of microstructure, not to mention the creation of anisotropic pore channels. Different from those solid natural plants, some liquid biomasses with inherent fluidity (e.g., proteins, enzymes, and polysaccharides) are in essence assembled by various small molecules through chemical interaction, which grants them superior morphological and microstructural plasticity [26]. Salting out is one of the common processes to solidify these liquid biomasses, because some strong electrolytes with high solubility can integrate with them to destroy their hydration shells, and on the other hand, these electrolytes can further break the charge balance on their particles, finally causing them accumulate and precipitate in water [33]. This transition from liquid phase to solid phase not only offers a good opportunity to re-direct the assembly of these biomasses, but also favors the fabrication of multicomponent composites owing to the anchoring of abundant inorganic ions during the salting-out process [35]. Although salting-out effect provides infinite possibilities to shape the microstructure of biomass, no studies to date attempt to construct 3D macroporous carbon-based composites in this way, especially for those with anisotropic pore channels.

In this article, we demonstrate a new strategy to construct 3D macroporous carbon-based foams with directional pore channels, where salting-out protein assemblies from egg white are selected as the precursor for the first time. Although eggs are generally important protein source with high nutritional value for human being, in some food processing industries (i.e., baby food and cake baking), egg white may be removed and discarded, and thus it may also be utilized as the potential raw materials of different functional materials [36–38]. Thanks to the electrostatic interaction between egg protein and ammonium metatungstate (AM), metatungstate ions will be also involved into final protein assemblies and converted to WC_1−x_ nanoparticles through high-temperature pyrolysis. The content of WC_1−x_ nanoparticles can be easily manipulated by the dosage of AM. After optimizing the chemical composition, the final WC_1−x_/C foams with directional pore channels exhibit excellent EM absorption performance when exposed to incident waves parallel to the pore channels in terms of both broadband absorption (6.3 GHz) and strong reflection loss (-72.0 dB), surpassing the performance of many previously reported biomass-derived composites. Radar cross section (RCS) simulation also manifests that WC_1−x_/C foams have good directional dependence and excellent radar stealth performance in practical applications. Of note is that this method not only frees the dependence on unidirectional freezing technique, but also simplifies the regulation of chemical composition, and thus it will provide great help for the fabrication of high-performance EWAMs in the future.

## Experimental Section

A required amount of AM (0.12, 0.24, 0.36, and 0.48 g) was firstly dissolved in 10 mL of deionized water at room temperature, and then 10 g of egg white was added dropwise over a period of five minutes into the pre-prepared AM solution without stirring. The resultant suspension rich in many white hollow microspheres was magnetically stirred with 300 r min^−1^ for 3 h at room temperature, and then inactivated at 90 °C for 10 min to ensure complete denaturation of proteins. After natural cooling to room temperature, the intermediate solid precursor was collected using a high-speed centrifuge, washed several times with deionized water, and freeze-dried for 72 h. The as-prepared precursor was transferred into a porcelain boat and pyrolyzed in a horizontally tubular furnace under Ar atmosphere at 450 °C for 0.5 h and 800 °C for 3 h, respectively. The heating rates from room temperature to 450 °C and from 450 to 800 °C were 3.0 and 5.0 °C min^−1^, respectively. Four final products were denoted as WCC-1, WCC-2, WCC-3, and WCC-4, respectively, with the increase in AM dosage. The other details of this work including materials characterization, electromagnetic parameter measurement, CST simulation and COMSOL simulation were available from the supporting information in the Springer Online Library.

## Results and Discussion

### Preparation and Structure Characterizations of WC_1−x_/C Foams

**Fig. 1:**
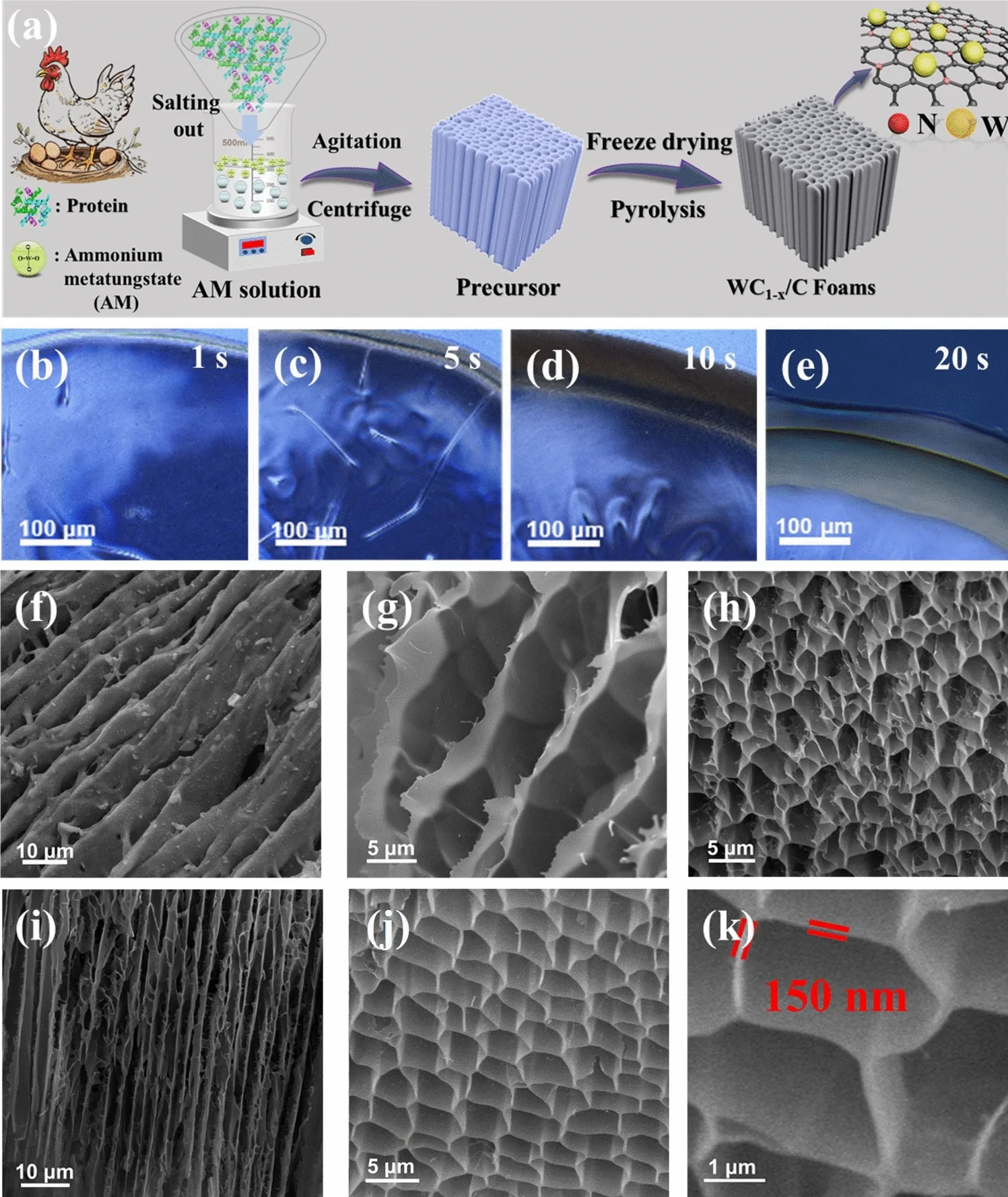
**a** Schematic preparation procedure of WC_1−x_/C foams. **b-e** microscopic images of protein salting-out processes at different time. **f** SEM image of assembled protein flakes. **g, h** SEM images of the precursor (i.e., assembled protein flakes after centrifugation). **i-k** SEM images of WCC-2

Figure 1a schematically illustrates the whole fabrication process of WC_1−x_/C foams with 3D directional pore channels. In general, egg white is slightly soluble in water, and thus when egg white is added dropwise in deionized water, it will settle at the bottom (Fig. S1a). Even after intense stirring, only a temporary suspension can be obtained, which will then return to its initial state (Fig. S1b). It is very interesting that in the presence of AM, the droplets of egg white will become quite different. When the dosage of AM is 0.06 g (Fig. S2a), only the aggregate of egg white is harvested, but there will be macroscopic vesicles once AM dosage exceeds 0.12 g (Fig. S2b, c), indicating the salting-out degree is very important for the subsequent self-assembly process of protein. Optical microscope monitors that as the reaction time extends, the salting-out effect will continuously thicken the walls of these vesicles (Fig. 1b-e), but these vesicles cannot support themselves very well, and most of them will spontaneously collapse and turn into salted protein flakes (Fig. S2d). Magnetic stirring will accelerate this process, and the resultant protein flakes can assemble themselves in a face-to-face manner through electrostatic interaction (Fig. 1f) [39, 40]. After high-speed centrifugation, the intermediate product preserves the directional arrangement of protein flakes, and more importantly, the thickness of these flakes is remarkably reduced, generating an open skeletal structure (Fig. 1g, h). This situation benefits from the removal of water under the action of centrifugal force. Of encouraging is that high-temperature pyrolysis does not destroy the arrangement of these flake-like units, but only induces an expected thermal shrinkage, and the pore size ranges from about 1.0 to 7.0 μm, which is a little smaller than that of directional protein flakes (Fig. 1i, j). A close inspection reveals that the thickness of final carbon flakes is about 150 nm (Fig. 1k). In addition, SEM images of WCC-1, WCC-3, and WCC-4 are also provided in Fig. S3, and these images identify that AM dosage not only impacts the salting-out process of egg white, but also affects the final structure of WC_1−x_/C foams. For example, as compared with WCC-2 (Fig. 1i, j), WCC-1 has relatively larger pore size, and there are many fractured areas (marked with red circles, Fig. S3a, b). From its cross section, one can also find some fused large pores. These results suggest that the low dosage of AM cannot ensure complete assembly of salting-out protein flakes, and thus some residual vesicles result in partial ultra-large pores. By comparison, WCC-3 gives relatively uniform macropores (Fig. S3c, d), indicating that high-dosage AM is helpful for the assembly of salting-out protein flakes. However, when the usage amount of AM increases excessively, the order of the structure in WCC-4 will be destroyed to some extent and many very large pores can be observed again (marked with white circles, Fig. S3e, f). The formation of more WC_1−x_ nanoparticles may the primary reason to induce the structure collapse due to the reaction between W species and carbon frameworks. At the same time, the aggregation of WC_1−x_ nanoparticles also makes the surface of carbon flakes rougher, and some aggregates are easily visible on their surface.

**Fig. 2:**
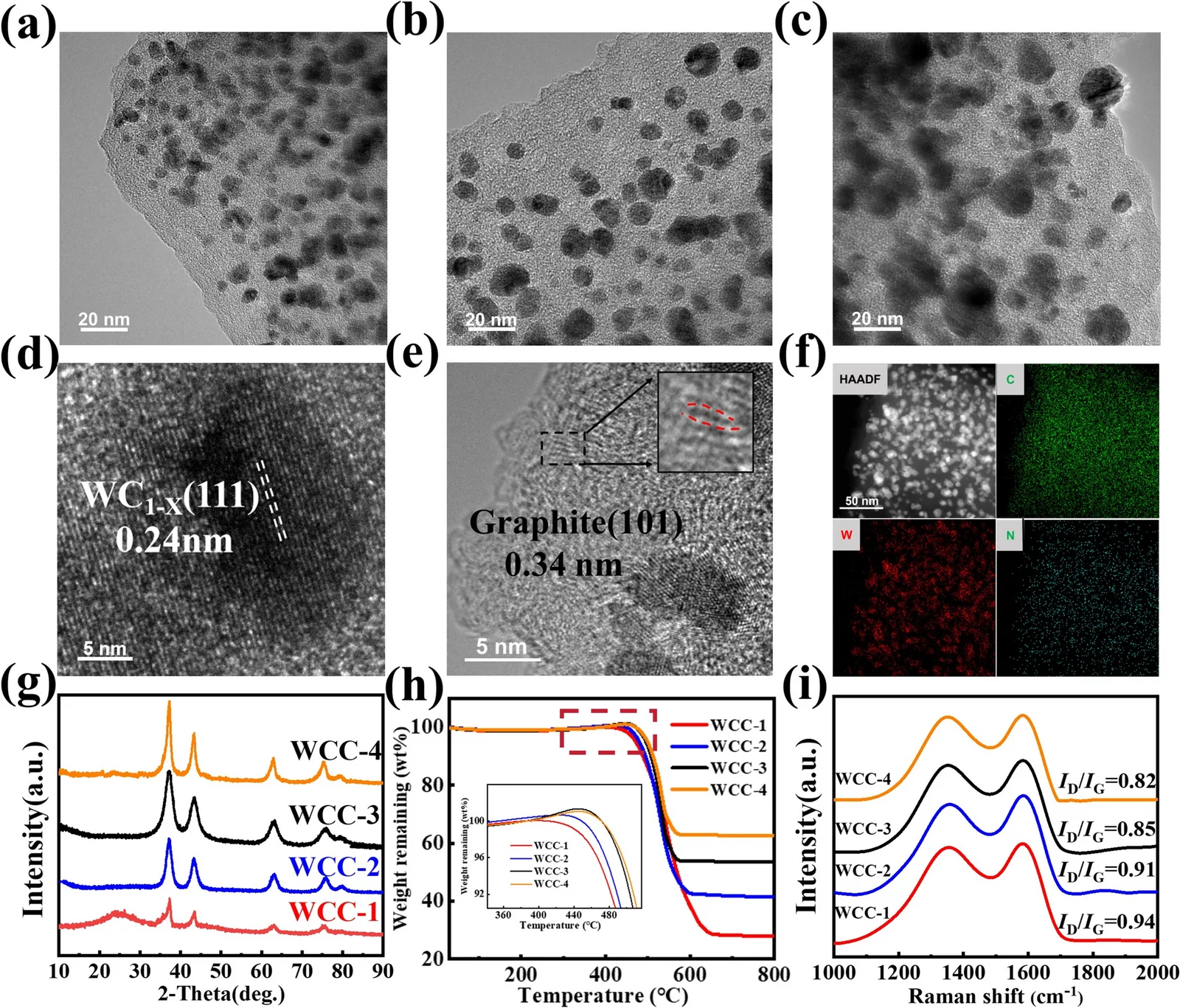
TEM images of **a** WCC-1. **b** WCC-2. **c** WCC-3. **d, e** HR-TEM images of WCC-2. **f** the corresponding element mapping images of C, W, N from WCC-2. **g** XRD patterns. **h** TG curves (inset is the local enlargement of the red rectangle). **i** Raman spectra

TEM technique is further employed to investigate the distribution of W-related species in final carbon-based foams. Some small nanoparticles less than 10 nm are observed to uniformly disperse on the surface of carbon flakes in WCC-1 (Fig. 2a), suggesting that W-related species have been successfully introduced during the salting-out process. With the increase in AM dosage, however, one can easily identify the growth of these nanoparticles, and they even have a noticeable reunion when the dosage reaches 0.36 g (Fig. 2b, c). When the dose of AM further increases to 0.48 g, the agglomeration phenomenon will be further aggravated in WCC-4, and even the edges of WC_1−x_ nanoparticles can no longer be completely distinguished (Fig. S4). High-resolution TEM image recognizes that the lattice fringe of these nanoparticles is about 0.24 nm (Fig. 2d), which is pretty close to that of (111) plane of WC_1−x_ with a typical face-centered cubic structure [41]. As is well known, tungsten carbide is a typical dielectric loss-type EWAMs, and thus WC_1−x_ nanoparticles can also participate in the attenuation of incident EM waves through conductive loss to some extent. However, by considering the difference in dielectric properties of WC_1−x_ nanoparticles and carbon flakes, the contribution of WC_1−x_ to conductive loss is relatively weak, and it mainly creates profitable interfacial polarization to strengthen the consumption of EM energy [42]. Furthermore, the introduction of WC_1−x_ nanoparticles not only introduces a large number of heterogeneous interfaces but also significantly optimizes the overall impedance matching of the composite material, avoiding the reflection of incident EM waves at the transmission interface and allow EM wave to enter the foams. In addition, another sinuous and discontinuous lattice fringe with a spacing of 0.34 nm is also recorded in TEM image (Fig. 2e). This observation serves as the evidence for the formation of tiny graphitic domains during high-temperature process [43]. However, current temperature is far away from the point for complete graphitization, and thus the frameworks of carbon foams are still amorphous in overall. These tiny graphitic domains not only facilitate the electron transfer when leakage current is induced by the applied EM field, but also contribute to interfacial polarization through the uneven distribution of space charges between graphitic domains and amorphous frameworks [44]. EDS elemental mapping further confirms the homogeneous dispersion of WC_1−x_ nanoparticles (Fig. 2f), and meanwhile, the result also uncovers the presence of abundant N atoms, which are from the initial egg white [45]. It is widely accepted that N atoms have stronger electronegativity than C atoms, and therefore, when N atoms substitute partial C atoms, they will also promote the electron redistribution and play as the sites to accomplish dipole orientation polarization [6]. All these results validate that the as-prepared WC_1−x_/C foams have many merits to reinforce the attenuation of incident EM wave.

Figure 2g shows XRD patterns of WC_1−x_/C foams from different AM dosage. As observed, all samples exhibit quite similar characteristic diffraction peaks at 2*θ* values of 37.2°, 43.1°, 62.2°, and 74.3°, which are precisely matched with (111), (200), (220), and (311) planes of face-centered cubic tungsten carbide (PDF#20–1316), respectively [46]. The absence of discernible characteristic diffraction peaks of metallic W or WO_3_ in XRD patters indicates that metatungstate ions attached on initial protein flakes have been completely converted into WC_1−x_ nanoparticles. Interestingly, WCC-1 also gives a broad and weak peak centered at 23.8°. This is a typical peak from amorphous materials, corresponding to the prediction of TEM result about the amorphous nature of carbon frameworks [47]. With the content increase in WC_1−x_ nanoparticles, this peak is concealed in other samples. Based on Scherrer’s equation [48], the average particle sizes of WC_1−x_ nanoparticles in WCC-1, WCC-2, WCC-3, and WCC-4 are estimated as 4.0, 6.0, 8.0, and 14.0 nm, respectively, again verifying the growth of WC_1−x_ nanoparticles with the increase in AM dosage. Kurlov and Gusev ever proposed a parabolic function of cell parameter (*a*) to indicate specific carbon content in cubic WC_1−x_ through the following equation [49]:

1 $$a = 0.4015 + 0.0481\left( {1 - x} \right) - 0.0236\left( {1 - x} \right)^{2}$$

With these measured patterns, the average *a* value of WCC-1, WCC-2, WCC-3, and WCC-4 are deduced as 0.424, 0.426, 0.425, and 0.425, respectively. The almost identical calculation results suggest the change of AM dosage just affects the content of WC_1−x_ nanoparticles, and thus the influence of the specific composition on WC_1−x_ nanoparticles can be excluded when different EM properties are discussed.

TG analysis is utilized to determine the content of WC_1−x_ nanoparticles in these carbon-based foams, because carbon frameworks can be easily burned off at high temperature under air atmosphere and WC_1−x_ nanoparticles will be converted WO_3_ with constant weight (Fig. S5**)**. As depicted in Fig. 2h, four samples display similar profiles of TG curves, including a region of slight weight gain and a region of remarkable weight loss. The weight gain benefits from the oxidation of WC_1−x_ nanoparticles, and the weight loss is attributed to the combustion of carbon frameworks. In terms of the residual weight (*R* wt%), the relative carbon content (*C* wt%) within the composites can be calculated by the following equation:

2 $$R_{wt\% } = \left( {1 - C_{wt\% } } \right)\frac{{M_{{{\mathrm{WO}}_{3} }} }}{{M_{{{\mathrm{WC}}_{{1 - {\mathrm{x}}}} }} }}$$

where *M* (WO_3_) and *M* (WC_1−x_) refer to the formula weight of WO_3_ and WC_1−x_, respectively. The results manifest that the relative carbon contents in WCC-1, WCC-2, WCC-3, and WCC-4 are 78.5, 67.4, 57.9, and 50.1 wt%, respectively. That is to say, the corresponding contents of WC_1−x_ nanoparticles can be roughly determined 21.5, 32.6, 42.1, and 49.9 wt%, respectively, which means that the chemical composition of WC_1−x_/C foams can be easily manipulated by AM dosage at the salting-out stage. It should be pointed out that the onset for the weight loss is slightly decreased from WCC-1 to WCC-4 (Fig. 2h, inset). In theoretical, WCC-1 has the smallest and least WC_1−x_ nanoparticles, and thus the oxidation of WC_1−x_ nanoparticles in WCC-1 should be completed earliest among the four samples and the combustion of its carbon frameworks will also occur firstly. The contradiction between the experimental results and the theoretical predictions is possibly raised from the different bonding state of carbon atoms in these composites. In this context, Raman spectra of these samples are further analyzed (Fig. 2i). All samples give two quite similar bands at 1350 cm^−1^ (D band) and 1590 cm^−1^ (G band), where D band generally arises from the *A*_1g_ symmetric vibrational mode, primarily originating from defective sites within the carbon matrix, and G band typically corresponds to the stretching of in-plane bonds of sp^2^ hybridized carbon atoms, usually in associated with graphitic sites [22, 49, 50]. Interestingly, there are no obvious differences in both the widths and Raman shifts, and thus their relative intensity ratios, *I*_D_/*I*_G_, may an effective pathway to discern the difference in carbon frameworks [22, 51]. Ferrari and Robertson ever recorded the changes of *I*_D_/*I*_G_ value from tetrahedral amorphous carbon (over 85% of *sp*^3^ sites) to perfect graphite in detail [52], and proclaimed the significantly different change in *I*_D_*/I*_G_ value between amorphous and graphitic carbon materials. They pointed out that in amorphous carbon materials, the formation of tiny graphitic domains generated a negligible impact on the intensity of G band, but the presence of six-fold rings would harden the vibrational density of states and decrease the disorder of bond angle/bending, thus resulting in a slight increase in the intensity of D band. Carbon frameworks in all WC_1−x_/C foams are still amorphous overall, and therefore, the gradual decrease in the value of *I*_D_*/I*_G_ suggests that the graphitic domains in carbon frameworks are progressively reduced from WCC-1 to WCC-3, which may be attributed to the fact that the reaction between metatungstate ions and carbon frameworks brings a number of defect sites. For the sake of discussion, we introduce the concept of relative graphitization degree to describe the content of graphitic domains in carbon frameworks, and the aforementioned results also means the relative graphitization degree of carbon frameworks is gradually weakened from WCC-1 to WCC-3. This conclusion is consistent with the prediction from TG analyses. As reported in previous studies, the decrease in the relative graphitization degree of carbon frameworks will be favorable for the improvement on impedance matching to some extent [14, 42, 53].

**Fig. 3:**
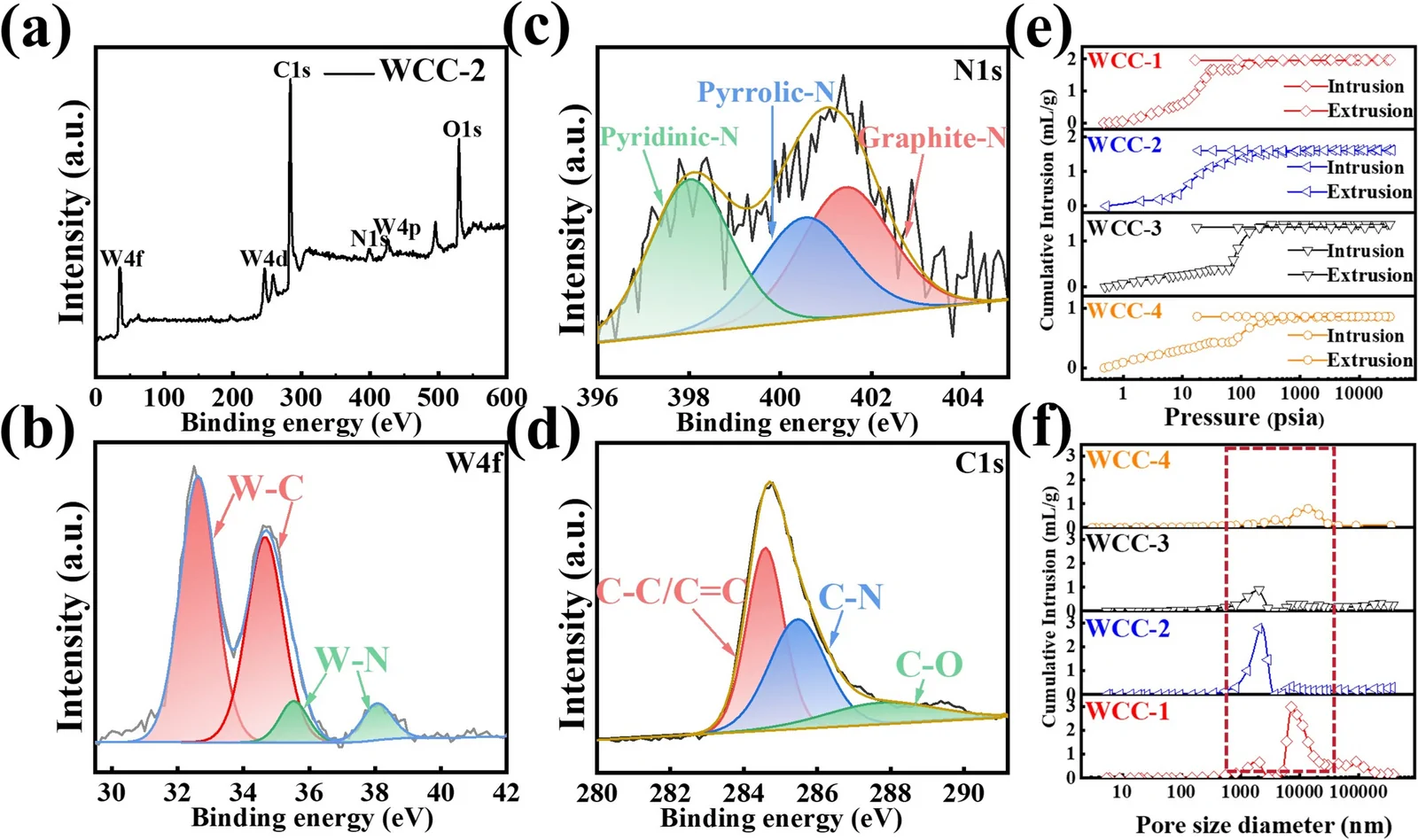
**a** XPS survey spectrum. **b** W 4*f* spectrum. **c** N 1*s* spectrum. **d** C 1*s* spectrum of WCC-2. **e** Mercury intrusion–extrusion isotherms. **f** Pore size distributions of WC_1−x_/C foams

XPS measurement is utilized to further elucidate the surface chemical composition and valence state of WC_1−x_/C foams. As depicted in Fig. 3a, the survey spectrum distinctly identifies prominent signal peaks of C, O, W, and N within WCC-2. The presence of O element may be from the surface oxidation, O-containing groups, or physically adsorbed small molecules. Notably, W 4*f* spectrum can be fitted into four peaks (Fig. 3b), where the two intense peaks with the binding energies at 32.5 and 34.5 eV can be attributed to W–C bond, and the other two characteristic peaks located at binding energies of 35.5 and 38.0 eV usually belong to W–N bond [54]. The absence of W–O bond implies that the carbon layer encapsulates WC_1−x_ nanoparticles densely and suppresses their oxidation effectively. The generation of W–N bond is commonly observed in WC_1−x_/C foams [54]. which is possibly induced by the fact that N-containing small molecules released from egg white are captured by W-related species and finally converted into N atoms filled in the interstitial sites of W atoms. In addition to a small amount of N atoms bonded with W atoms, N 1*s* spectrum also records three kinds of different N configurations in carbon frameworks (Fig. 3c), i.e., pyridinic-N (398.6 eV), pyrrolic-N (400.5 eV), and graphitic-N (401.3 eV) [14, 55]. The nitrogen sources for doping carbon atoms originate from both the biologically rich nitrogen inherent from the protein itself and the ammonium ions present in AM. In general, pyridinic-N and pyrrolic-N are the two primary states of N elements present in all nitrogen-containing composites, and graphitic-N peak fitted in WC_1−x_/C foams indicates an enhancement in the relative graphitization degree at high temperature. As illustrated in Fig. 3d, C 1*s* spectrum of WCC-2 can be fitted into the distinct signal peaks, corresponding to the binding energies of 284.6, 285.5, and 287.8 eV, which are attributed to C–C/C = C, C–N, and C = O groups, respectively. Both doped nitrogen atoms and oxygen-containing functional groups are widely regarded as active sites to generate dipolar orientation polarization in previous studies [55, 56].

Although N_2_ adsorption–desorption technique is commonly utilized to acquire textural information of porous materials, it is not applicable to the pores whose size is over 50 nm. Therefore, we herein employ mercury intrusion porosimetry to accurately uncover the difference in the microstructure of different WC_1−x_/C foams. It is worth noting that solid materials typically do not exhibit strong affinity to mercury, and thus the intrusion of mercury into a pore is strongly dependent on the assistance of external pressure, and the smaller size the pore has, the higher the external pressure is required [56, 57]. One can see that with the increase in external pressure, all four samples indeed present obvious increase in mercury intrusion (Fig. 3e). However, their saturated intrusion volume and intrusion behavior are significantly distinguishable. For example, WCC-1 reaches the saturated mercury intrusion (2.0 mL g^−1^) at the pressure of 500 psi, and more importantly, it realizes more than 82% of mercury intrusion at the pressure of 29 psia. This phenomenon suggests that WCC-1 has many extra-large pores that can be easily accessible by liquid mercury. In contrast, WCC-2 only achieves 33% of mercury intrusion at the pressure of 60 psia, and presents an intrusion jump within the pressure range of 60–310 psia, which means that the pore size in WCC-2 is relatively uniform. WCC-3 exhibits similar mercury intrusion behavior, but its saturated intrusion volume is further reduced to 0.9 mL g^−1^ due to its higher content of WC_1−x_ nanoparticles. With the further increase in the dose of AM, the directional porous structure is difficult to maintain at high temperatures. The moderate collapse of the porous structure enables mercury intrusion to reach saturation at a lower pressure. Meanwhile, due to the further increase in the content of WC_1−x_ nanoparticles in WCC-4, its saturated mercury intake also decreases to 0.84 mL g^−1^. Pore size distribution further reveals the difference among these WC_1−x_/C foams (Fig. 3f). WCC-1 has two most probable distributions in pore size, which span from 1.0–4.0 and 5.0–15.0 μm, respectively, and their peaks are centered at *ca.* 2.0 and 8.2 μm, respectively. For WCC-2, the probable distribution around 1.0–4.0 μm is greatly enhanced, and the one around 5.0–15.0 μm is drastically reduced. As indicated by SEM images, the ordered degree of porous structure in WCC-2 is remarkably improved, and thus the former pore size distribution is undoubtedly from the directional arrangements of carbon flakes, and the latter is derived from the vesicles of protein assemblies that are not completely salted out. WCC-3 gives quite similar pore size distribution, but its intensity is obviously declined, indirectly suggesting that the porous structure has been moderately destroyed with the increase in WC_1−x_ nanoparticles. As for WCC-4, its pore size distribution is increased to 7.0–20.0 μm, which correspond SEM images very well (Fig. S3e, f). Based on the curves of mercury intrusion porosimetry, the porosities of WCC-1, WCC-2, WCC-3, and WCC-4 are 57.5%, 56.1%, 54.7%, and 52.4%, respectively. Such close porosities indirectly indicate that WC_1−x_ nanoparticles are inclined to attach on the surface of carbon flakes and do not aggregate together to block the pores, that is to say, the loading of WC_1−x_ nanoparticles will create positive contribution through interfacial polarization. Although the porosity of the sample shows a gradually decreasing trend with the increase inWC_1−x_ nanoparticles, previous studies have proved that there is no strict qualitative relationship between porosity and EM wave absorption performance. The existence of porous structures mainly plays a role in optimizing the impedance matching of composites. However, more WC_1−x_ nanoparticles still increase the density of the foams, and the corresponding surface areas of WCC-1, WCC-2, WCC-3, and WCC-4 are deduced as 3.3, 2.5, 2.0, and 1.7 m^2^ g^−1^, respectively.

### EM Absorption Performance of WC_1−x_/C Foams

**Fig. 4:**
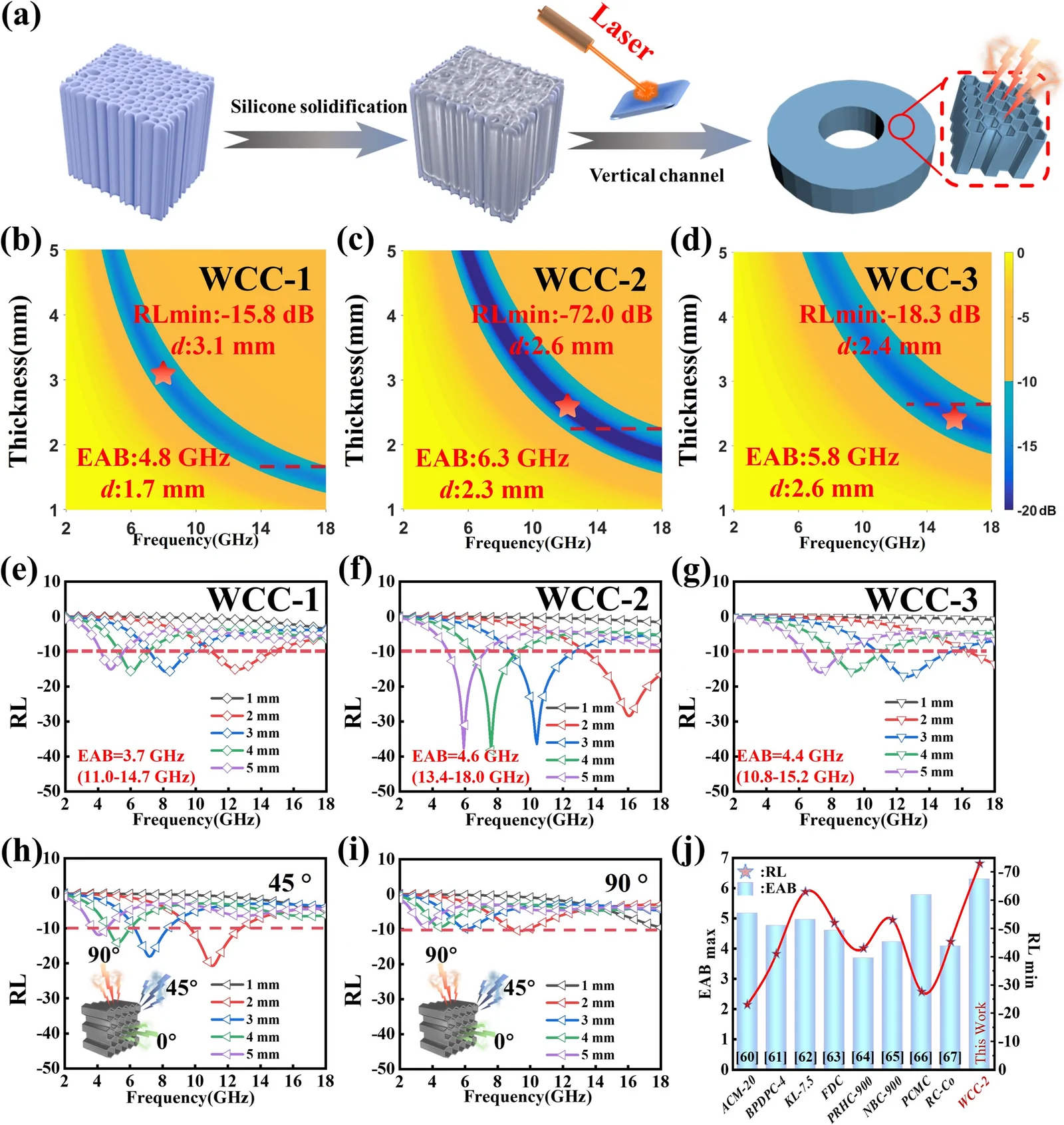
**a** Schematic diagram on EM test. 2D RL maps of **b** WCC-1. **c** WCC-2. **d** WCC-3. RL curves with some specific thicknesses of **e** WCC-1. **f** WCC-2. **g** WCC-3. RL curves of WCC-2 with some specific thicknesses from the incident angle of **h** 45°. **i** 90°. **j** Comparison in RL_min_ and EAB with those reported EWAMs derived from biomass

As we mentioned above, 3D macroporous carbon-based foams with directional pore channels have been reported as advanced EWAMs in previous studies, but when their EM absorption performance is measured, most studies should break them and then press the mixture of fragments and paraffin into a customized ring [58]. In this way, the advantages of directional pore channels will be compromised, because it is impossible to orient the pore channels of fragments in the same direction. To accurately present the contribution of directional pore channels to EM absorption, we herein solidify the monoliths of WC_1−x_/C foams into silicone matrix, and then cut the resultant composite into the standard size (DN: 7.0 mm, D: 3.0 mm, height: 2.0 mm) for measurement (Fig. 4a, please find the details in the supporting files). The reasons for using silicone as the organic binder can be explained from the following aspects. First, silicone is also a wave-transparent medium that will not bring additional EM attenuation. Second, silicone can be cured quickly at room temperature, which makes the operation relatively simple. Third, silicone can endow WC_1−x_/C foams with good toughness, and thus WC_1−x_/C foams cured with silicone will not be broken during laser cutting process. As the filling amount (*R*%) of WC_1−x_/C foams in silicone matrix, it can be determined by the following equation:

3 $$R\% \, = M_{1} /M_{2}$$

where *M*_1_ and *M*_2_ are the mass of WC_1−x_/C foams before and after being cured with silicone. The 2D RL maps of WC_1−x_/C foams with the frequency (2.0–18.0 GHz) of EM wave and the thickness (1.0–5.0 mm) of EWAMs as two independent variables. For clarify, the strongest RL value is artificially cut off at − 20.0 dB. It is clear that all these samples can attenuate incident EM wave to some extent, but their specific performance is still distinguishable. For example, WCC-1 exhibits its minimum RL value of − 15.8 dB at 8.0 GHz with the thickness of 3.1 mm (Fig. 4b), and it can produce obvious absorption performance in the frequency range of 4.1–18.0 GHz by manipulating the thickness from 1.0 to 5.0 mm (Fig. S6). The largest EAB of WCC-1, a frequency range where RL is no more than − 10.0 dB, is defined as 4.80 GHz with the thickness of 1.7 mm. Compared with WCC-1, WCC-2 gives similar applicable frequency range (4.8–18.0 GHz), while its minimum RL value (− 72.0 dB) is much lower than that of WCC-1, and more importantly, its largest EAB is significantly extended to 6.3 GHz (Fig. 4c). It is unfortunate that WCC-3 fails to further upgrade its performance, and its minimum RL value and largest EAB fall back to − 18.3 dB and 5.8 GHz (Fig. 4d), respectively, and what’s worse, its applicable frequency range is also diminished to 6.3–18.0 GHz when the thickness varies from 1.0 to 5.0 mm (Fig. S6). As the increase in WC_1−x_ nanoparticles, WCC-4 gives drastic degradation in EM absorption performance, and its largest EAB and minimum RL directly drop to 4.8 GHz and -12.7 dB, respectively (Fig. S7). For the purpose of a more intuitive comparison, we have plotted their RL curves with some specific thicknesses (Figs. 4e-g and S8). One can find that no matter how much the thickness is set, WCC-2 always produce stronger RL value and broader EAB than other three samples, demonstrating its superior EM absorption performance. Of note is that we also adjust the direction of laser cutting to make EM wave propagate vertical to the pore channels and oblique to the pore channels with an angel of 45° and 90°. The results indicate that the change of the incident direction indeed induces the degradation of EM absorption performance (Fig. 4h, i), validating that the importance of the oriented channels for the attenuation of incident EM wave. In addition, we also summarize the minimum RL value and the largest EAB of WCC-2 and some typical biomass-derived carbon-based foams in previous studies (Fig. 4j) [60–67]. One can easily confirm the advantages of WCC-2 in EM absorption performance. Even compared with some representative carbides/carbon composites, WCC-2 still shows significant superiority in RL intensity and response bandwidth (Table S1) [20, 21, 68–71]. The results clearly highlight that WCC-2 can be taken as an extremely competitive candidate for high-performance EWAMs, which also proclaims that the conversion of salting-out proteins will be an attractive method to prepare directional carbon-based foams.

### EM Parameters and Absorption Mechanisms of WC_1−x_/C Foams

**Fig. 5:**
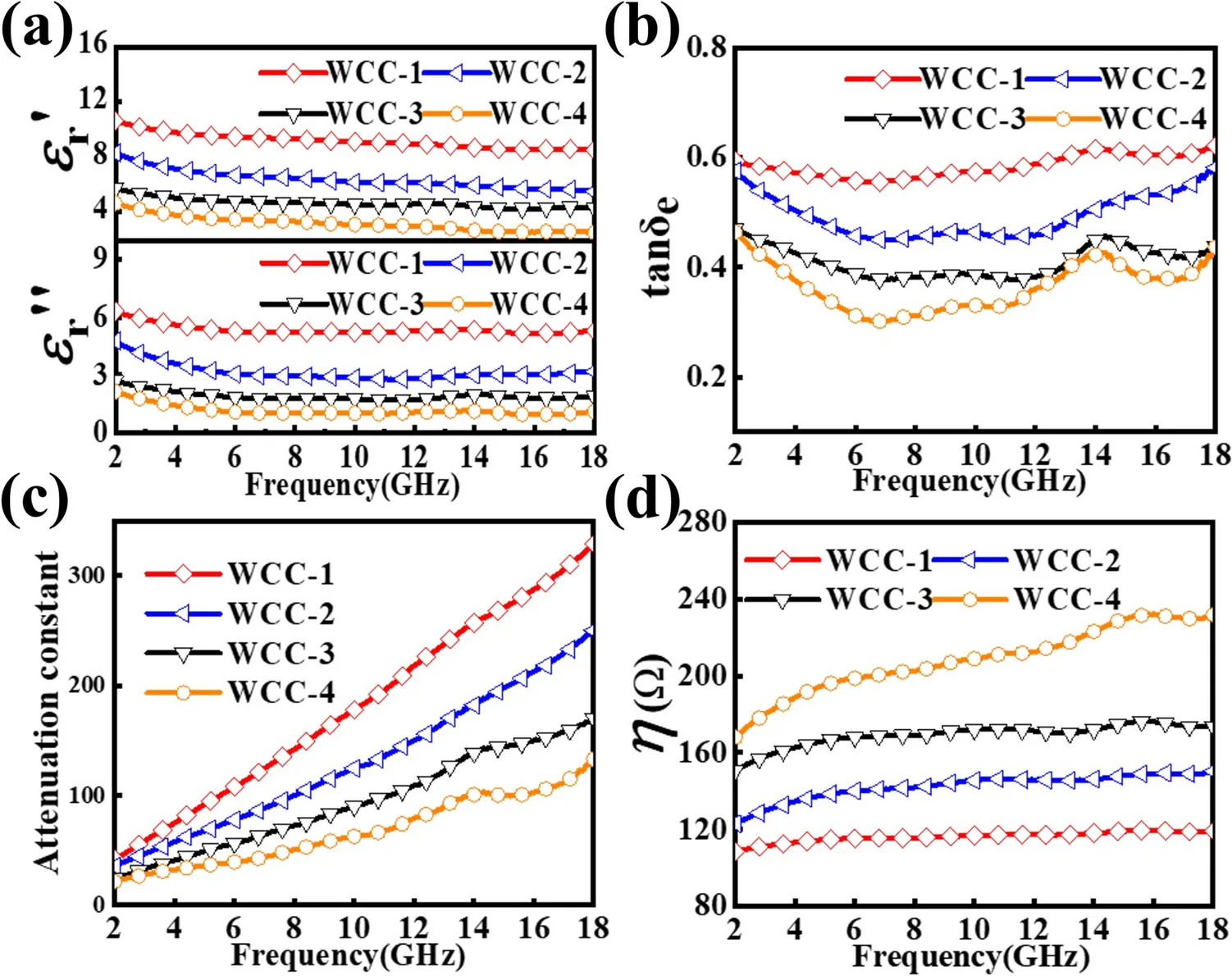
**a** Real parts (*ε*_r_*'*) and imaginary parts (*ε*_r_*"*) of complex permittivity. **b** Dielectric loss tangent (tan*δ*_e_). **c** Attenuation constant. **d** Wave impedance (*η*) of WC_1−x_/C foams

It is well-known that under the given frequency and thickness, the EM parameters, specifically relative complex permittivity (*ε*_r_ = *ɛ*_r_′-j*ɛ*_r_″) and relative complex permeability (*μ*_r_ = *μ*_r_′-j*μ*_r_″), are the dominant intrinsic factors to determine the absorption performance of EWAMs according to transmission line theory [66]. However, WC_1−x_/C foams in our case do not contain any magnetic components, and thus their real parts and imaginary parts of relative complex permeability are all constants pretty close to 1 and 0, respectively (Fig. S9), which indicates that dielectric loss is the only possible pathway for them to dissipate EM energy. Figure 5a presents frequency-dependent *ɛ*_r_′ and *ɛ*_r_″ curves of different WC/C foams in 2.0–18.0 GHz. Both of *ɛ*_r_′ and *ɛ*_r_″ curves of these samples exhibit typical frequency dispersion effect, especially in the frequency range of 2.0–8.0 GHz, which usually serves as an indication of dipolar orientation polarization, arising from the rotation of dipoles lagging behind frequency variation [70]. As aforementioned, carbon frameworks in WC_1-x_/C foams are overall amorphous, and thus there are many defective sites in carbon flakes, and in cooperation with abundant N atoms and O-containing functional groups, numerous polarization centers will be generated under an applied EM field, and thus dipolar orientation polarization can be taken as a potential pathway to dissipate EM energy. However, *ɛ*_r_′ and *ɛ*_r_″ curves of these samples do not continuously decrease across the entire frequency range, and their reduction degrees are almost negligible, and even for WCC-2, WCC-3, and WCC-4, their *ɛ*_r_″ curves give slight increases when the frequency is over 14.0 GHz. Such an abnormal increase in *ɛ*_r_″ curve is ordinarily attributed to interfacial polarization in previous studies [71]. There are two kinds of heterogeneous interfaces in our system, the interface between WC_1-x_ nanoparticles and carbon frameworks, as well as the interface between WC/C foam and silicone. It is well-known that the contribution of polarization loss can be described by the series connection of capacitors and resistors [72], and the relationship between the polarization contribution to relative complex permittivity and the distribution parameters of the circuit is deduced as the following equation:

4 $$j\omega \varepsilon_{0} \varepsilon_{r} = \frac{1}{{\frac{1}{{\sigma_{s} }} + \frac{1}{{j\omega \kappa_{s} }}}}$$

where *σ*_s_ and *K*_s_ are the conductivity and the capacitance of the series resistor, respectively. By considering that the conductivity of silicone is quite far behind that of EWAMs, the equivalent conductivity *σ*_s_ at the interface of EWAMs/silicone can be ignored, which means that the polarization contribution therein is also negligible. In contrast, WC_1-x_ nanoparticles are generally regarded to have Pt-like properties [20], and thus their conductivity will be much higher than that of silicone. That is to say, the interfacial polarization contributive to the attenuation of incident EM wave is primarily dependent on the uniform dispersion of WC_1-x_ nanoparticles along with the whole carbon frameworks. To better visualize the occurrence of polarization, the curves of *ɛ*_r_′ *vs ɛ*_r_″ are further plotted in Fig. S10. Debye dielectric theory clearly represents the relationship between *ε*_r_′ and *ε*_r_" with the following equation [73, 74]:

5 $$\left( {\varepsilon_{r}^{\prime } - \frac{{\varepsilon_{s} - \varepsilon_{\infty } }}{2}} \right)^{2} + \left( {\varepsilon_{r}^{\prime \prime } } \right)^{2} = \left( {\frac{{\varepsilon_{s} - \varepsilon_{\infty } }}{2}} \right)^{2}$$

where *ε*_s_ and *ε*_∞_ are the static permittivity and dielectric constant at infinite frequency, respectively. According to this equation, if one polarization relaxation process occurs in a dielectric medium, the curve of *ɛ*_r_′ *vs ɛ*_r_″ will present a semicircle, i.e., Cole–Cole semicircle, which describes the polarization relaxation behavior of a dielectric medium with a single relaxation time under alternating electric fields [74]. As shown in Fig. S10, one can find that there are at least three semicircles in each curve, which forcefully validates the occurrences of multiple polarization relaxations [75, 76]. However, of note is that apart from these semicircles, all four samples give quasi-linear tails with much longer dielectric span than those semicircles, implying that conductive loss produce superior contribution to polarization loss [77]. In principle, from WCC-1 to WCC-4, the relative graphitization degree of carbon frameworks is gradually degraded and the loading amount of WC_1-x_ nanoparticles is gradually increased, and thus the overall effects of various polarization relaxations are correspondingly enhanced. However, the values of *ɛ*_r_′ and *ɛ*_r_″ do not follow this prediction, and instead, both of them gradually decrease with increasing the loading amount of WC_1-x_ nanoparticles. For instance, at the frequency point of 10.0 GHz, the *ɛ*_r_′ and *ɛ*_r_″ values for WCC-1, WCC-2, WCC-3, and WCC-4 are 9.1 and 5.2, 6.2 and 2.3, 4.6 and 1.8, 3.1 and 1.0, respectively. This order means that conductive loss is the primary mechanism for EM dissipation in WC_1-x_/C foams, and polarization loss plays an auxiliary role in this process. The reason for strong conductive loss of WCC-1 can be explained by two aspects: on one hand, carbon frameworks of WCC-1 have relatively high graphitization degree, which facilitates electron transfer and ensures stronger energy consumption when leakage current is induced, and on the other hand, the electrical conductivity of WC_1-x_ nanoparticles is far less than that of carbon frameworks, thus the less WC_1-x_ nanoparticles, the stronger conductive loss. Dielectric loss tangent (tan δ_e_ = *ɛ*_r_″/*ɛ*_r_′) is a common parameter to evaluate the contribution of total dielectric loss (Fig. 5b) [70]. The results reveal that tan δ_e_ values of these samples have the same order with those of *ɛ*_r_′ and *ɛ*_r_″ values, manifesting that conductive loss is in charge of dielectric loss. Of note is that within high-frequency range, all tan δ_e_ curves exhibit some fluctuations, which typically come from the relaxation resonances and again confirm the contribution from polarization effects [53].

Attenuation constant (*α*) is a parameter that can elucidate the amplitude attenuation of EM wave in a transmission medium, while it can also reflect the total attenuation ability of EWAMs intuitively. With the measured EM parameters, *α* values at different frequency points can be deduced in terms of the following equation:

6 $$\alpha = \frac{\sqrt 2 \pi f}{c} \times \sqrt {\left( {\mu_{r}^{^{\prime\prime}} \varepsilon_{r}^{^{\prime\prime}} - \mu_{r}{\prime} \varepsilon_{r}{\prime} } \right) + \sqrt {\left( {\mu_{r}^{^{\prime\prime}} \varepsilon_{r}^{^{\prime\prime}} - \mu_{r}{\prime} \varepsilon_{r}{\prime} } \right)^{2} + \left( {\mu_{r}{\prime} \varepsilon_{r}^{^{\prime\prime}} + \mu_{r}^{^{\prime\prime}} \varepsilon_{r}{\prime} } \right)^{2} } }$$

Figure 5c represents the frequency-dependent *α* values of different WC_1−x_/C foams. All samples give monotonously increased *α* values from 2.0 to 18.0 GHz, indicating that the attenuation of EM wave within high-frequency range will be relatively easy for them as compared with the attenuation of low-frequency EM wave. Interestingly, at any a point in the frequency interval, the order of *α* values is highly consistent with those of *ɛ*_r_′ and *ɛ*_r_″ values, as well as that of tan δ_e_ values. This high consistency further manifests that dielectric loss is the overwhelming pathway for the consumption of EM energy. However, one can also find that WCC-1 with the strongest dielectric loss and attenuation ability does not produce the best EM absorption performance among the four samples, and instead, WCC-2 with inferior dielectric loss and attenuation ability displays better performance. This situation implies that EM absorption performance is not solely determined by the attenuation ability of EWAMs. The concept of characteristic impedance is more important for EWAMs than attenuation ability in many cases [28, 30]. If the gap in characteristic impedance between free space and EWAMs is too large, most of EM wave cannot go through the interface smoothly, resulting in the strong reflection of EM wave at the transmission interface [28, 30]. In that case, no matter how strong attenuation ability EWAMs have, their absorption performance is still weak because the proportion of incident EM wave is too limited. In an effort to reveal the reason for the relatively poor performance of WCC-1, we employ the concept of wave impedance (*η*) to evaluate the matching degree of characteristic impedance, and *η* values are calculated by the following equation:

7 $$\eta = \sqrt {\frac{{\mu_{{\mathrm{r}}} }}{{\varepsilon_{{\mathrm{r}}} }}}$$

As shown in Fig. 5d, *η* values of these four samples display gradual upward trend with increasing frequency, but the increments are more distributed in low-frequency range. The average *η* values for WCC-1, WCC-2, WCC-3, and WCC-4 are 115, 147, 170, and 200 Ω, respectively. It is well known that the characteristic impedance of free space is approximately 377 Ω. From this point, WCC-1 creates the largest difference in the characteristic impedance as compared with that of free space, WCC-1 has the poorest matching degree of characteristic impedance. Therefore, the relatively weak EM absorption performance of WCC-1 can be attributed to its mismatched characteristic impedance. By comparison, although WCC-2 has inferior attenuation ability to WCC-1, the matching degree of characteristic impedance between WCC-2 and free space has been moderately amended, which further results in the enhancement of EM absorption. As for WCC-4, its high *η* values means that the propagation of EM wave across the transmission interface is allowable, but its low *α* values suggest incapable of dissipating incident EM energy, and thus it also fails to generate comparable EM absorption performance to WCC-2. These analyses manifest that the balance between attenuation ability and characteristic impedance is the key point for the design of high-performance EWAMs.

**Fig. 6:**
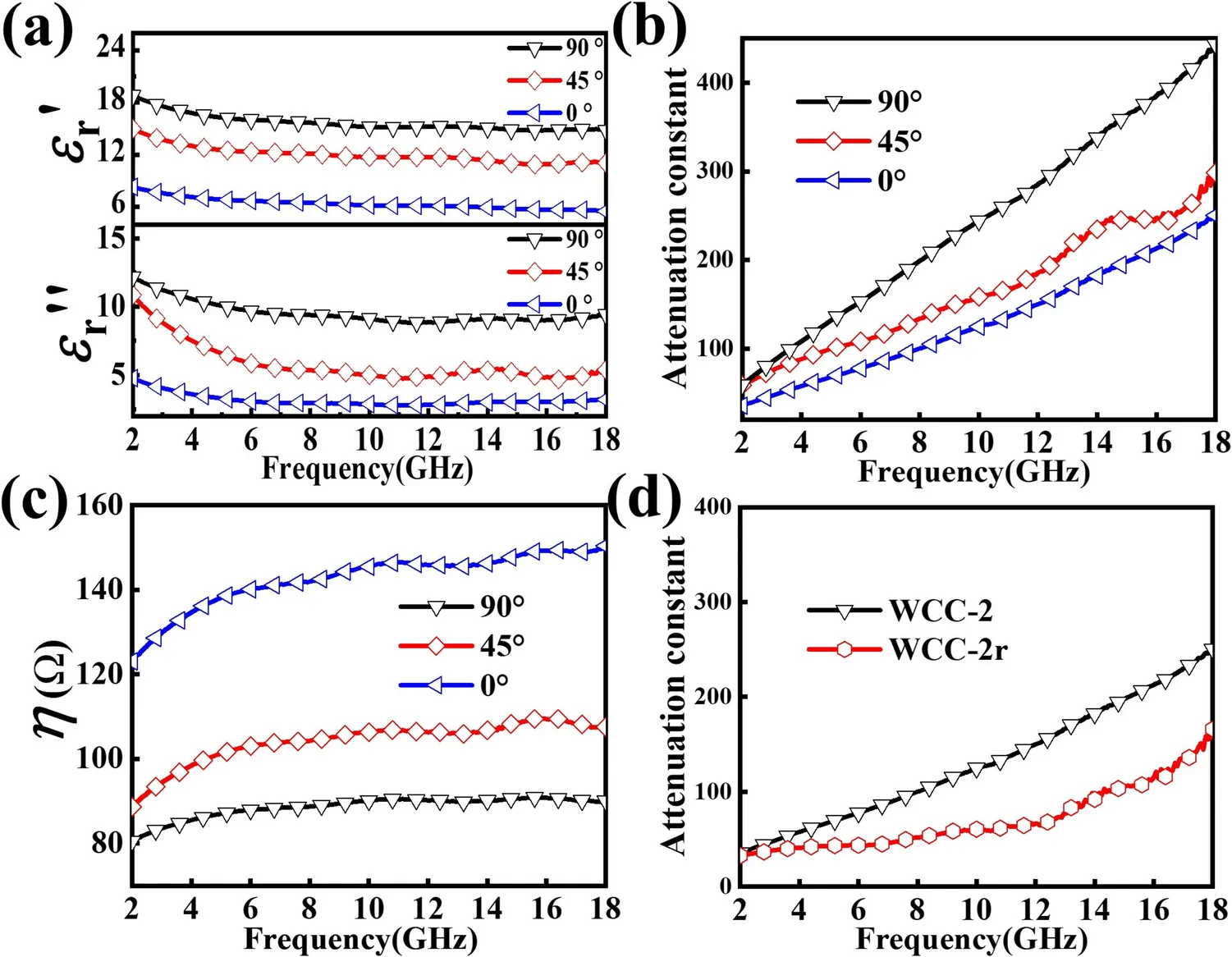
**a** Real parts (*ε*_r_*'*) and imaginary parts (*ε*_r_*"*) of complex permittivity. **b** Attenuation constant (*α*). **c** Wave impedance (*η*) of WCC-2 with different incident angles. **d** Attenuation constant (*α*) of WCC-2 and WCC-2r

To disclose the directional dependence of EM absorption, the relative complex permittivity of WCC-2 measured from different incident angle is further provided in Fig. 6a. One can clearly observe that as the direction of incident EM wave changes from parallel to the channel (0°) to perpendicular to the channel (90°), both *ε*_r_*'* and *ε*_r_*"* will be drastically increased. Correspondingly, *α* values and *η* values have also undergone significant changes (Fig. 6b, c), where *α* values increase with the incident angle and *η* values decrease with the incident angle. These results mean that the increase in incident angle will strengthen the overall loss ability of WCC-2, but weaken its matching degree of characteristic impedance [30]. This is because when EM wave propagates along the directional pore channels, the extremely high porosity will provide considerable convenience for its incidence, while the high porosity also poses a significant obstacle for electron transfer. As a result, the loss ability in this case is relatively weak. With the increase in incident angle, the porosity at the incident cross section will be gradually reduced, which increases the difficulty in the propagation of EM wave, and especially for the perpendicular incidence, it is equivalent to the illumination of EM wave on an intact carbon flake, and the good conductivity of this intact carbon flake will cause strong reflection of EM wave. Therefore, even if this intact carbon flake has very strong attenuation ability, it still fails to afford the consumption of EM energy. In another words, the directional dependence of EM absorption is essentially caused by a trade-off effect between attenuation ability and impedance matching (Fig. 6b, c). Besides, we also obtain WC_1−x_/C foam with random macropores (WCC-2r) by disrupting the directional arrangement of protein flakes after centrifugation through magnetic stirring. It is interesting that WCC-2r is not assembled by uniaxially aligned carbon flakes any more, but consisted of disordered carbon fibers and bulkies (Fig. S11), confirming the damage of original structure. EM measurement reveals that WCC-2r has similar dielectric frequency dispersion behavior to that of WCC-2, while its *ε*_r_*'* and *ε*_r_*"* are obviously smaller than those of WCC-2 (Fig. S12). Correspondingly, the values of tan δ_e_ and *α* of WCC-2r are both smaller than those of WCC-2, suggesting that the dielectric loss ability in WCC-2r is degraded (Fig. 6d). This is because the absence of uniaxially aligned carbon flakes will bring difficulties to electron transfer, thus weakening the conductive loss, a dominant factor for dielectric loss. As a result, WCC-2r cannot promise comparable EM absorption performance to WCC-2, and its strongest RL and maximum EAB are − 13.8 dB and 3 GHz (Fig. S13), respectively. This phenomenon clearly highlights the importance of directional pore channels for the attenuation ability of WC_1−x_/C foams.

**Fig. 7:**
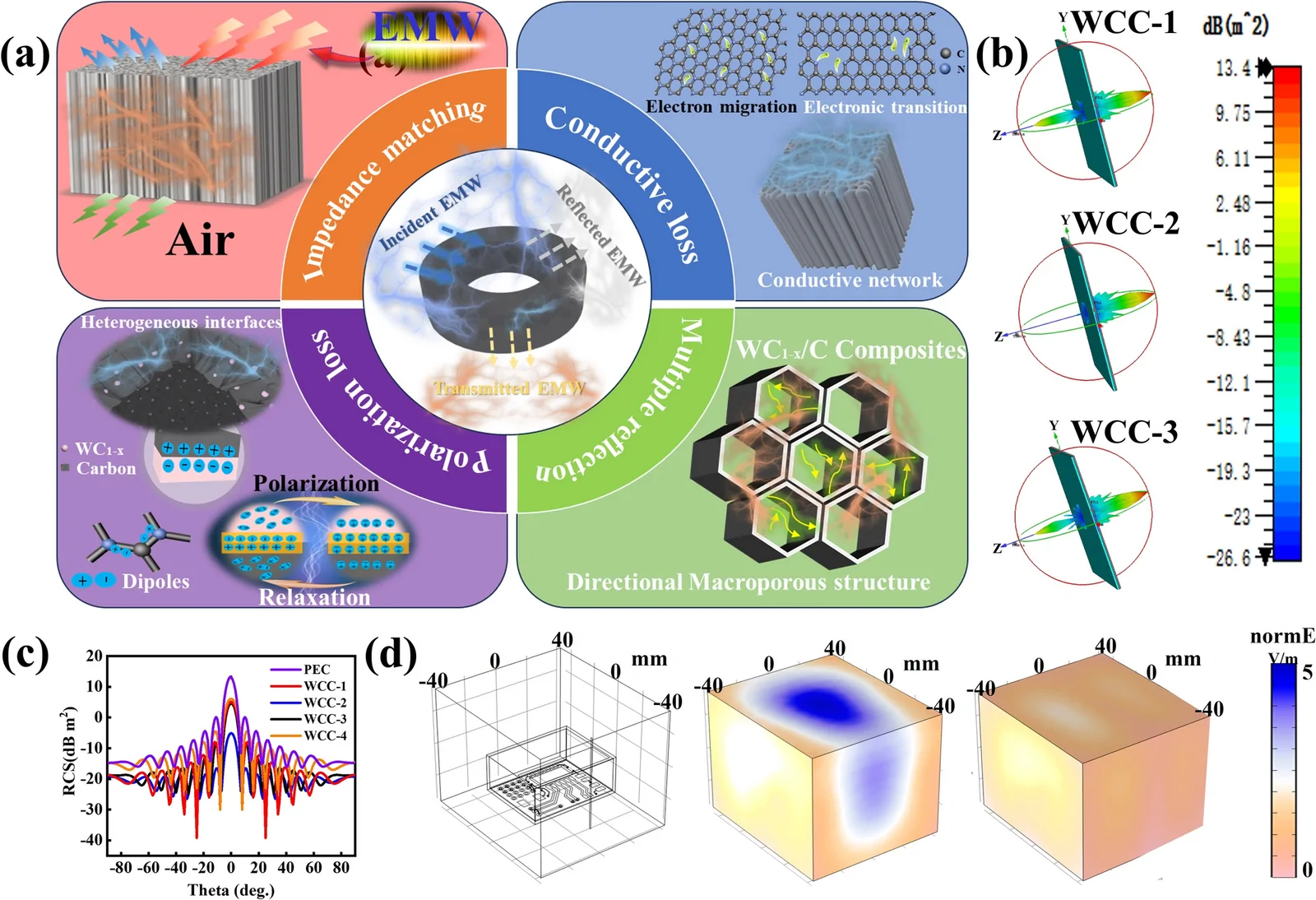
**a** Schematic illustration for the EM absorption mechanisms of WCC-2. **b** 3D radar wave scattering signal maps of PEC coated with WC_1−x_/C foams. **c** RCS in polar coordinate system of WC_1−x_/C foams. **d** Chip anti-interference stimulation with WCC-2

Based on the results above, the mechanisms for the excellent EM absorption performance of WCC-2 have been systematically summarized in Fig. 7a. First, the directional pore channels create a profitable transmission interface that facilitates the propagation of EM wave and reduces the probability of backward reflection, especially for EM wave parallel to pore channels. This situation suggests that WCC-2 will be particularly suitable for solving EM pollution irradiated from a specific direction. Second, as compared the carbon-based foam with disordered macropores, the uniaxially aligned carbon flakes favor electron transfer and hopping, thus generating strong conductive loss. Third, the salting-out process makes AM evenly anchored on protein flakes and then be converted into highly dispersed WC_1−x_ nanoparticles located on final carbon flakes. The difference in dielectric property between WC_1−x_ nanoparticles and carbon flakes will induce powerful interfacial polarization under alternating EM field. Moreover, as indicated by Raman spectra, the formation of WC_1−x_ nanoparticles brings rich defective sites, which in together with heteroatoms and functional groups in amorphous carbon flakes can consolidate dipolar reorientation polarization. Both of these two polarization effects will play as considerable supplements to dielectric loss. Another point needs to be mentioned is that WC_1−x_ nanoparticles have inferior electron transfer ability as compared with carbon flakes, and thus they can mediate electron transfer to some extent, which avoids characteristic impedance mismatching caused by strong leakage current along carbon frameworks. Finally, the formation of micron-level macroporous structure can promote the multiple reflection of incident EM wave, equivalent to extending its transmission distance, thus resulting in more energy consumption. Although classical physical principle perdicts that a pore or particle with much smaller size than incident wavelength cannot induce scattering or reflection behavior of EM wave [78], it is important to note that the effective counteraction against EM wave does not solely rely on an individual channel, but a macroscopic medium composed of numerous pores. This macroscopic medium with the same size of wavelength can be understood as an uneven medium compose of two phases: solid phase and pore phase, and thus EM wave bypasses this medium directly. Therefore, when EM wave goes through this uneven medium, the alternating solid phase and void phase are bound to cause the changes in the propagation behavior of EM wave. Once its propagation direction is changed some times, the so-called multiple reflection will occur, and this loss mechanism has also been approved in many previous studies [79].

Radar cross section (RCS) value is an intuitive parameter to evaluate the radar stealth capability of a layer with EWAMs under actual far-field conditions [16, 80, 81]. Computer Simulation Technology (CST) Microwave Studio is employed to acquire simulated RSC values of the WC_1−x_/C foams in this study, and the details about the model can be indexed in the supporting file. As shown in Fig. 7b, all WC_1−x_/C foams can produce noticeable reduction in RSC values as compared with perfect electric conductor (PEC), where WCC-2 gives the smallest RSC value among them, again demonstrating its good radar stealth capability (Figs. S14 and S15). The angular dependence of RSC value is further stimulated in the range of −90° to 90° (Fig. 7c). Although WCC-1 seems to display its superiority at some specific incident angles (e.g., 10°, 30°, 50°), its average value of RCS reduction (defined as the difference in RSC value PEC and PEC with EWAMs) is not the best and only deduced as 6.5 dB m^2^. In contrast, the average values of RCS reduction from WCC-2, WCC-3, and WCC-4 are 13.6, 7.3, and 6.8 dB m^2^, respectively. Such a situation means that the application of WCC-2 is not limited by parallel incidence, and even for EM wave from different direction, it can also be utilized as eligible EWAMs. Of note is that in addition to radar stealth application, EWAMs widely demonstrate their significant potential for protecting the normal operation of precision instruments. To prevent mutual interference between sensitive electronic components during operation, anti-interference coatings are typically applied to chip surfaces, but conventional EM shielding materials just block external EM wave and are incapable of addressing internally generated EM interference. In this context, we further stimulate the performance of WCC-2 as functional materials to alleviate EM interference among different chip units (the detailed information can be indexed in the supporting file, Fig. 7d, left). One can see that a bare chip unit will generate an obvious electric field intensity up to 5.0 V m^−1^ (Fig. 7d, middle). Interestingly, when a protection layer of WCC-2 with the thickness of 2.0 mm is coated, the electric field intensity will be drastically decreased less than 2.0 V m^−1^ (Fig. 7d, right). It is undoubted that the utilization of WCC-2 layer indeed suppresses EM leakage of the chip, which naturally reduces EM interference among different electronic components, validating the wide application prospects of WCC-2.

## Conclusions

Directional three-dimensional macroporous carbon foams decorated with WC_1−x_ nanoparticles have been successfully constructed through the self-assembly of salting-out protein and subsequent high-temperature pyrolysis. The unique microstructure of such carbon-based foams offers remarkable advantages in matching characteristic impedance and reinforcing EM loss ability. The in situ generated WC_1−x_ nanoparticles highly dispersed on carbon frameworks not only amend dielectric property of carbon foams, but also provides profitable interfacial polarization. The resultant WC_1−x_/C foams with rational composition can produce exceptional EM absorption performance, achieving strong reflection loss of -72.0 dB and broad effective absorption bandwidth of 6.3 GHz. Furthermore, CST and COMSOL simulations validate the enormous application potential of WC_1−x_/C foams in military stealth and anti-EM interference. This work provides a new idea to construct directional 3D carbon-based foams, making it no longer dependent on unidirectional freezing technique, and thus we believe this work will be helpful for the design and fabrication of advanced EM functional materials in the future. However, there also remain two important challenges in the approach of this research. On one hand, the foam size herein is at the centimeter level, and therefore, an improved strategy to fabricate carbon-based foams with larger size is desirable, and on the other hand, the pore size in the resultant foam is dependent on the self-assembly of protein flakes, and how to regulate the pore size rationally is still unclear.

## Supplementary Information

Below is the link to the electronic supplementary material.

Supplementary file1 (DOCX 4988 KB)
