# Supplementary material for: Directional Three-Dimensional Macroporous Carbon Foams Decorated with WC1−x Nanoparticles Derived from Salting-Out Protein Assemblies for Highly Effective Electromagnetic Absorption
Source: Nanomicro Lett. 2025 Sep 15;18:71. doi: 10.1007/s40820-025-01920-z (PMC12436266; doi:10.1007/s40820-025-01920-z)
Supplement: Supplementary file 1 — Supplementary file1 (DOCX 4988 KB) [file 40820_2025_1920_MOESM1_ESM.docx]

Supporting Information for

**Directional Three-Dimensional Macroporous Carbon Foams Decorated with WC_1-x_ Nanoparticles Derived from Salting-out Protein Assemblies for Highly Effective Electromagnetic Absorption**

Yongzheng Chen^1^, Lixue Gai^1^, Bo Hu^1^, Yan Wang^1^, Yanyi Chen^1^, Xijiang Han^1^, Ping Xu^1^ and Yunchen Du^1,^ ***

^1^ State Key Laboratory of Space Power-Sources, School of Chemistry and Chemical Engineering, Harbin Institute of Technology, Harbin 150001, P.R. China

*Corresponding author. E-mail: [yunchendu@hit.edu.cn](mailto:yunchendu@hit.edu.cn) (Yunchen Du)

**S1 Materials Characterization**

Powder X-ray diffraction (XRD) data were recorded on a Rigaku D/MAXRC X-ray diffractometer (45.0 kV, 50.0 mA) using Cu Kα source. Raman spectra were measured on a confocal Raman spectroscopic system (Renishaw, In Via) using a 633 nm laser. X-ray photoelectron spectroscopy (XPS, Kratos, ULTRA AXIS DLD) was recorded with monochrome Al Kα (1486.6 eV) radiation to study the surface states. The salting-out process of protein was obtained by Olympus BX53 fluorescence microscope (Olympus, Tokyo, Japan). Scanning electron microscopy (SEM) images of all samples were acquired by a HELIOS NanoLab 600i (FEI, USA). Transmission electron microscopy (TEM) images were obtained on a Tecnai F20 instrument operating with an accelerating voltage of 200 kV. An AutoPore IV 9510 porosimetry was used to carry out mercury injection porosimetry (MIP) measurement. The thermogravimetric analysis was carried out on a SDT Q600 thermogravimetric analyzer (TGA) in the temperature range from room temperature to 800 °C with a heating rate of 10 °C min^−1^ under air atmosphere. The relative complex permittivity and complex permeability in the frequency range of 2.0-18.0 GHz were measured using an Agilent N5234A vector network analyzer (Agilent, USA) for the calculation of refection loss characteristics. Prior to measurement, WCC-x was cured with silicone and subsequently sliced into rings with a thickness of 2.0 mm, an outer diameter of 7.0 mm, and an inner diameter of 3.0 mm.

**S2 Silicone Curing Process**

Firstly, 10 mL of pure silicone solution, 3 mL of diluent, and 1.5 mL of curing agent were measured separately and added into a beaker and stirred at room temperature for 30 minutes to ensure uniformity. Subsequently, the mixture was placed in a vacuum drying oven for multiple vacuum extractions to eliminate the air bubbles within the solution. When the bubbles were completely removed, the mixture was carefully dripped onto the sample surface until it was fully covered. The sample was then placed back into the vacuum drying oven for additional vacuum extraction to ensure the mixture penetrated thoroughly into the sample. This process was repeated 2–3 times to ensure no bubbles remained after vacuum extraction. Finally, the sample was cured at 60°C for 24 h to achieve the final product, which was subsequently processed using a laser cutting machine before undergoing performance testing.

**S3 CST Simulation**

The radar stealth characteristics in practical application of EWAMs were evaluated and predicted by simulating the radar cross section (RCS) using Computer Simulation Technology (CST) Microwave Studio. The constructed simulation model was consisted of two 180×180 mm^2^ square flat panels, where the lower layer was a perfect electric conductor (PEC) with a thickness of 1.5 mm and the upper layer was an EM wave absorption layer with a thickness of 2.0 mm. The measured EM parameters based on transmission line theory were imported and then fitted with the dispersion model to set the corresponding absorption layer. In the simulation process, the linear polarized plane EM wave direction was set for the positive direction of *Z* axis to the negative direction of *Z* axis. Meanwhile, the electric field and the magnetic field polarization propagation direction along *X* axis and *Y* axis, respectively. In addition, the type of solver was an integral solver and far-field open boundaries in all directions are to simulate the free space environment. Finally, we fixed the frequency of the emitted wave at 12.5 GHz during the simulation. The RCS values can be calculated by using the following equation:

where *S* and *λ* represent the area of the target object simulation model and the wavelength of the electromagnetic wave, respectively, and *E*_s_ and *E*_i_ refer to the electric field intensity of the transmitted wave and the electric field intensity of the received wave, respectively.

**S4 COMSOL Simulation**

The research about the potential application of EWAMs in other scenarios was completed by COMSOL Multiphysics software. Specifically, a circuit model with a copper layer covering its outer surface was utilized to simulate the operational state of a chip. The circuit board is made of FR4 material, and the printed circuit pattern is defined by the copper layer. To capture the loss caused by the limited electrical conductivity in the geometrically thin copper layer, we used the transition boundary condition and set the thickness of the conductive layer to 30 μm. Subsequently, we placed the chip in a 2mm thick ABS plastic casing. The outer wall of the housing was set as a perfect electric conductor (PEC), and WCC-*x* was employed as boundary conditions for the inner wall. After finishing these configurations, a square housing with an edge length of 80 mm was added externally. A scattering boundary condition was assigned to the external boundary to effectively absorb all emitted radiation and prevent secondary reflection of incident wave at the boundary. Finally, to calculate the leakage of EM wave, the time-averaged power flux was integrated over the external boundary by using an integral solver, enabling a quantitative assessment of EM leakage.

**Supplementary Figures and Tables**

**
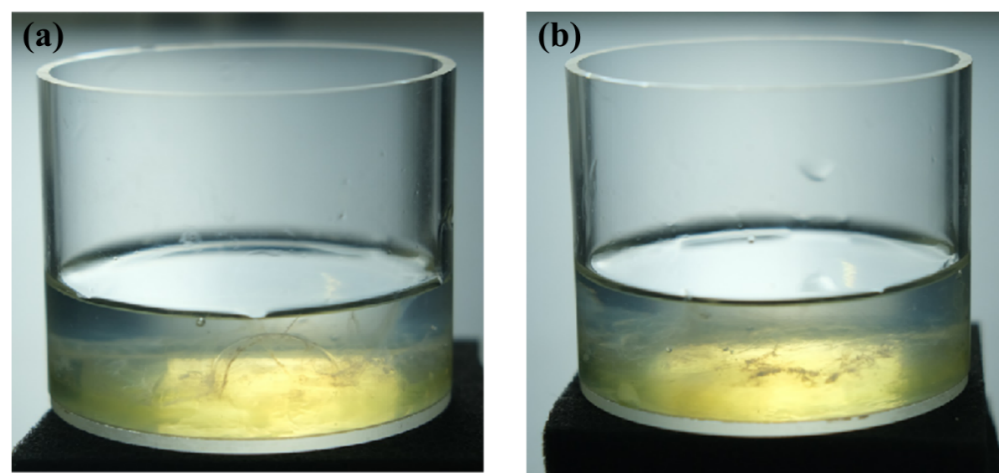
**

**Fig. S1** Optical images of egg white in deionized water **a** before stirring. **b** after stirring

**
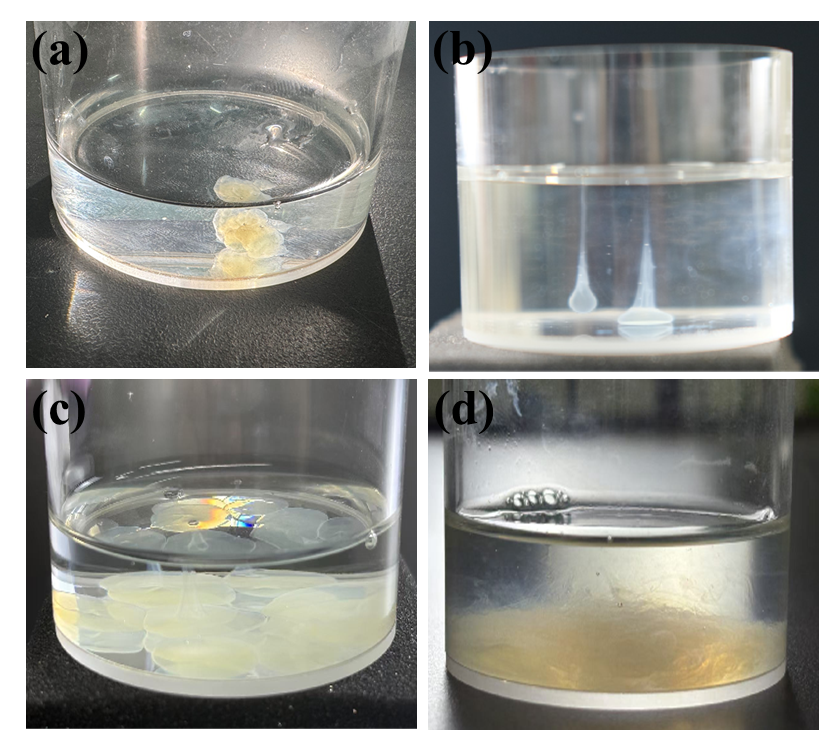
**

**Fig. S2** **a** Optical images of egg white in low-dose AM solution **b**, **c** Optical images of egg white in high-dose AM solution **d** Optical image after stirring in AM solution


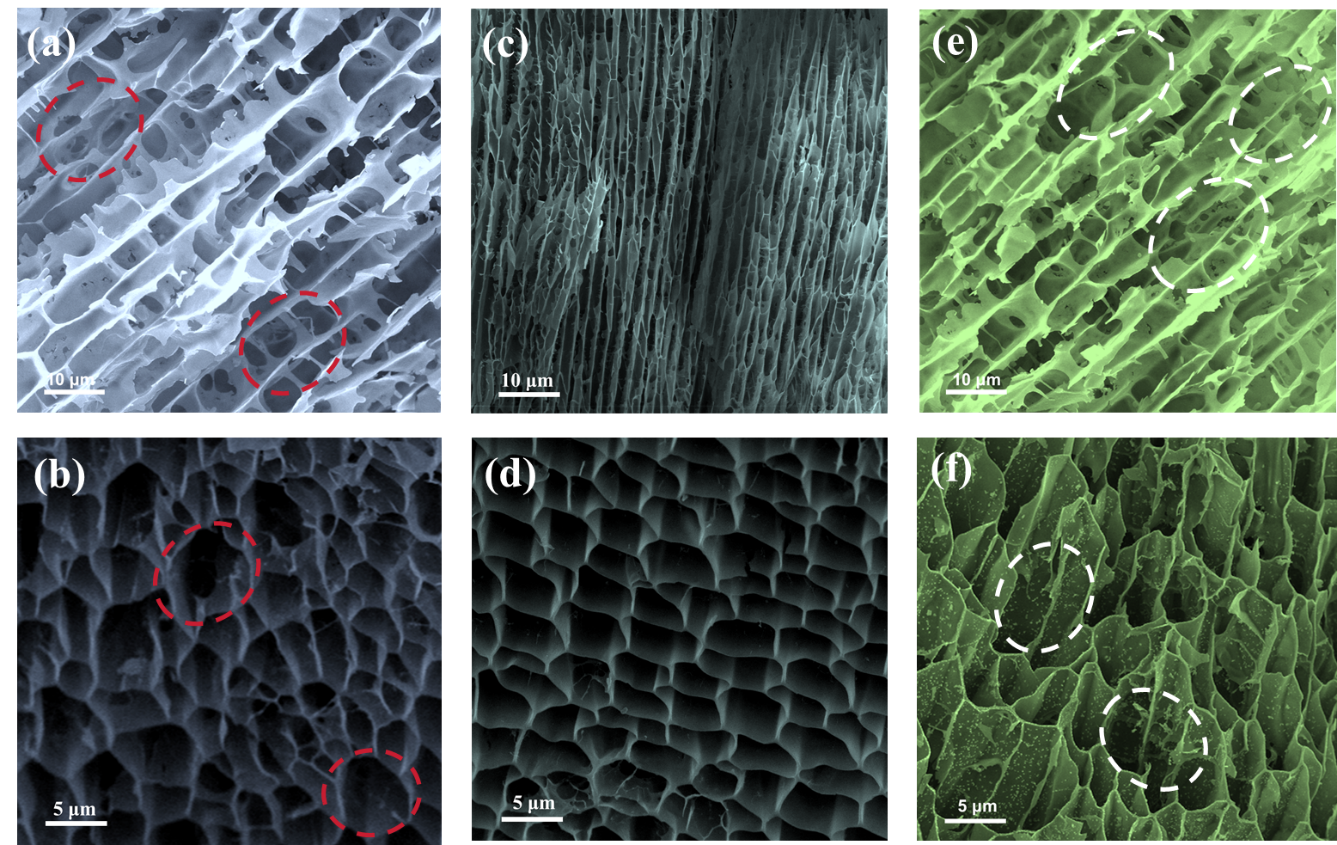


**Fig. S3** SEM images of **a, b** WCC-1 **c, d** WCC-3 **e, f** WCC-4


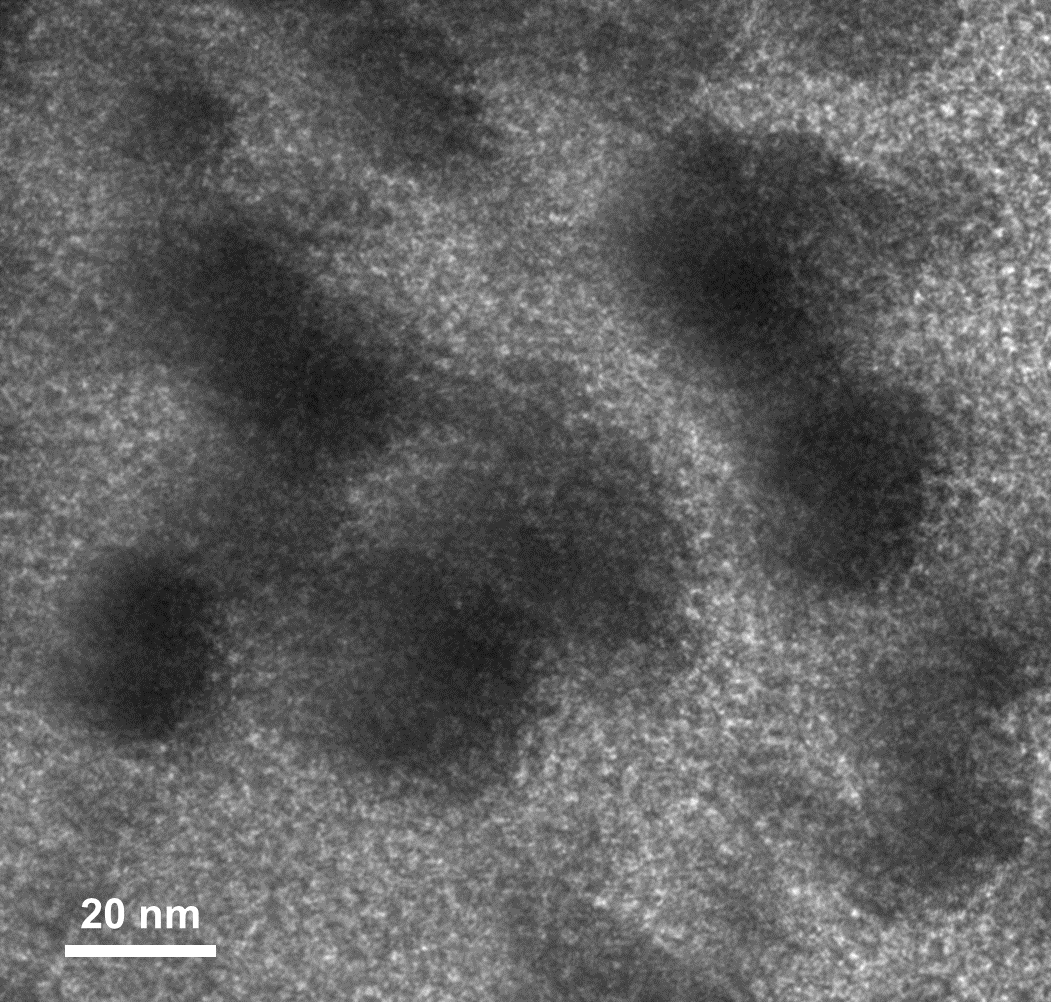


**Fig. S4** TEM images of WCC-4

**Fig. S5** XRD pattern of residual solid after TG measurement

**Fig. S6** Effective absorption ranges of WC_1-x_/C foams by accumulating the thickness from 1.0 to 5.0 mm


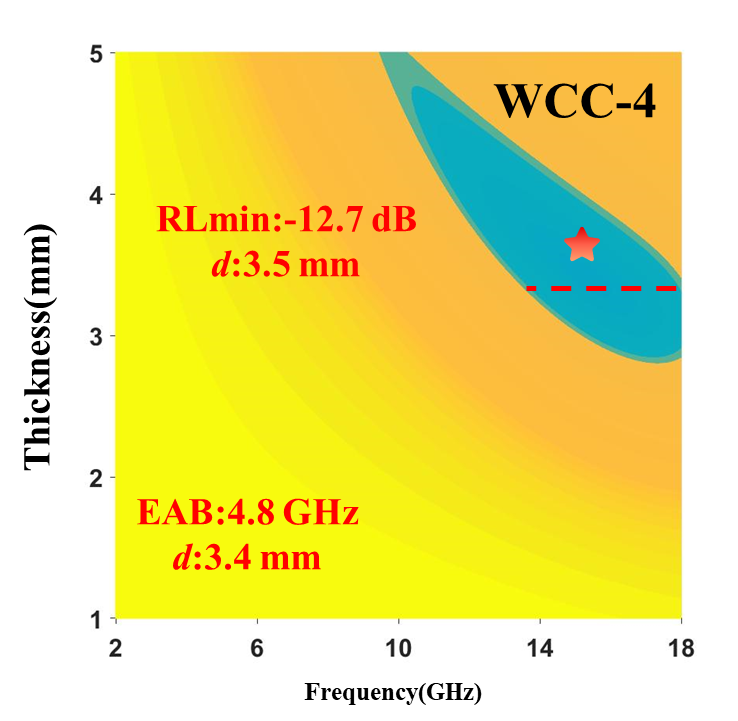


**Fig. S7** 2D RL mapping of WCC-4


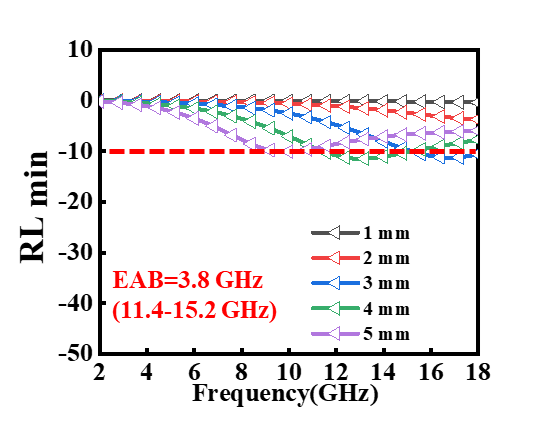


**Fig. S8** RL curves with some specific thicknesses of WCC-4

**
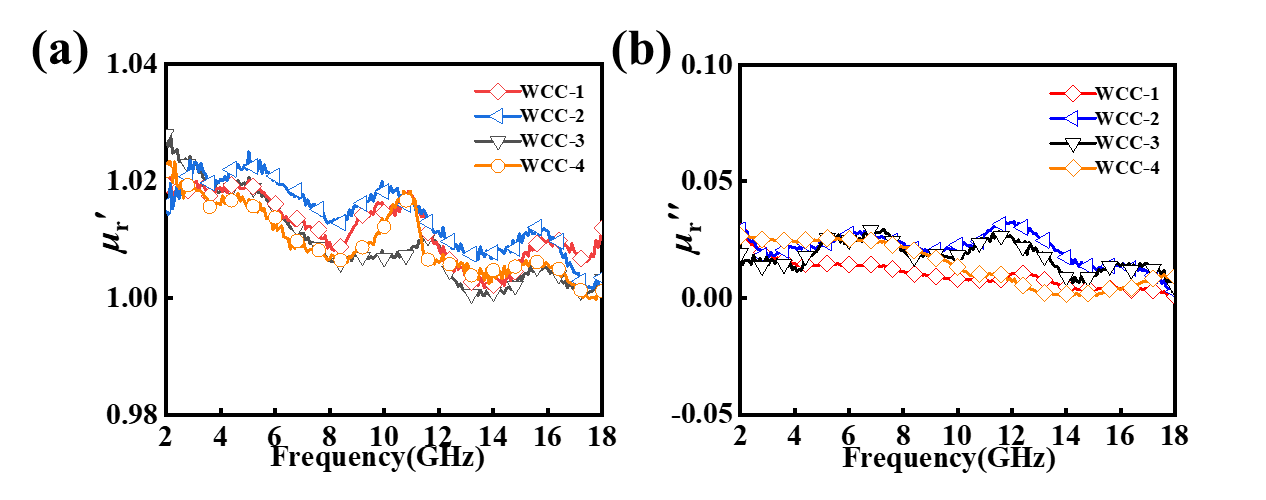
**

**Fig. S9** **a** Real parts (*μ*_r_*'*) and **b** imaginary parts (*μ*_r_*"*) of relative complex permeability of WC_1-x_/C foams


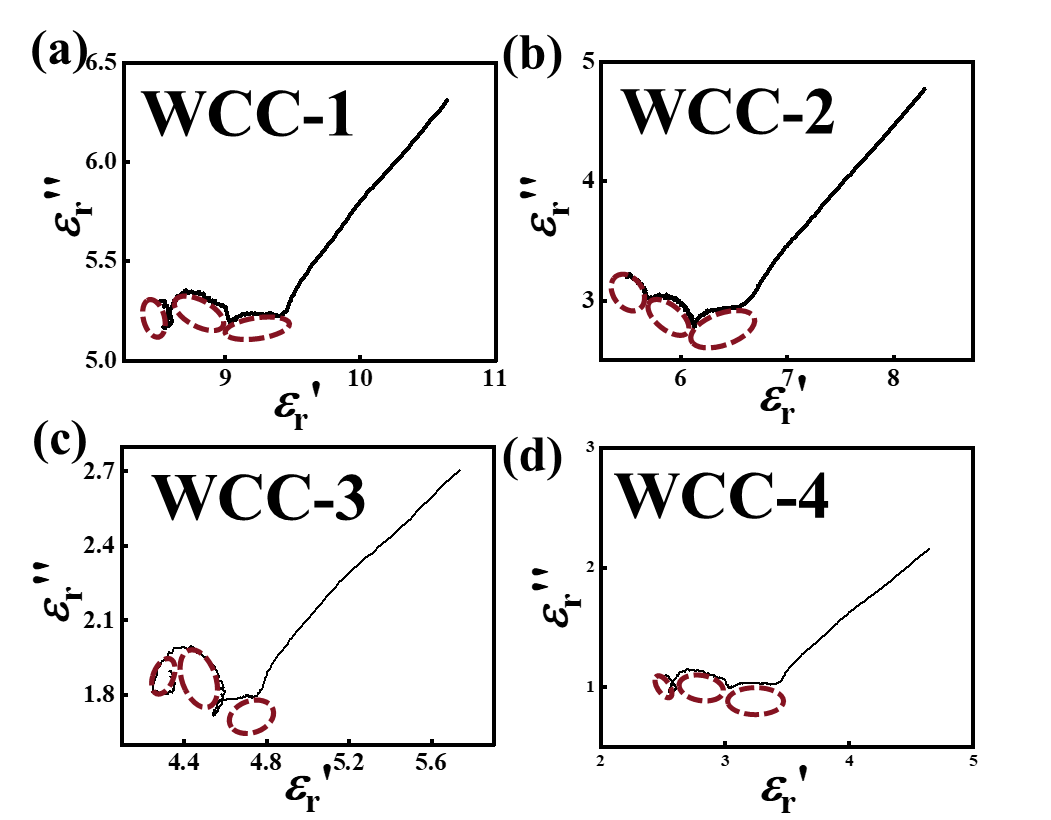


**Fig. S10** *ɛ*_r_′ *vs.* *ɛ*_r_″ curves of **a** WCC-1. **b** WCC-2. **c** WCC-3 d WCC-4


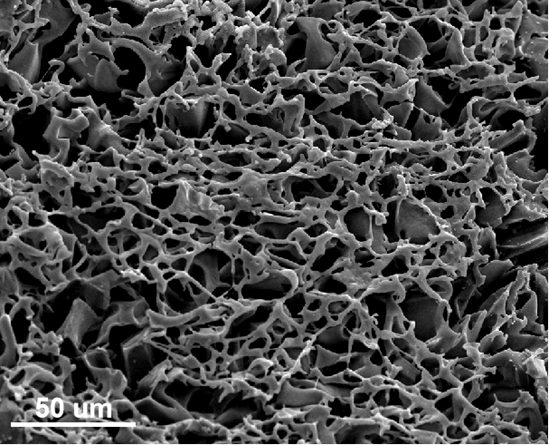


**Fig. S11** SEM image of WCC-2r


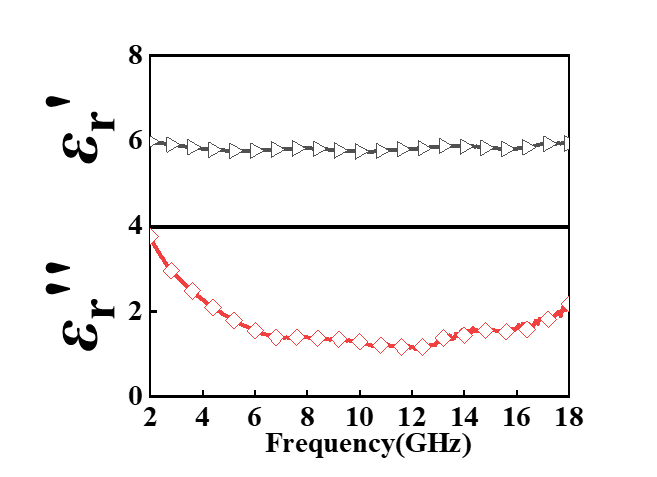


**Fig. S12** Real parts (*ε*_r_*'*) and imaginary parts (*ε*_r_*"*) of complex permittivity of WCC-2r


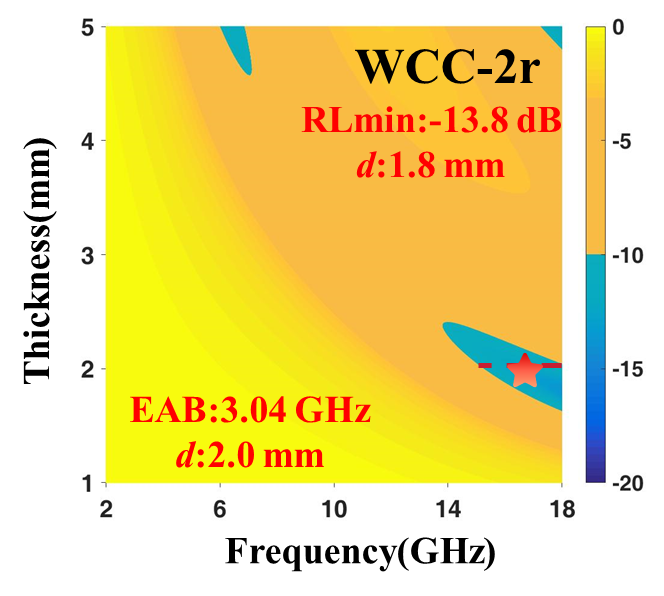


**Fig. S13** 2D RL mapping of WCC-2r


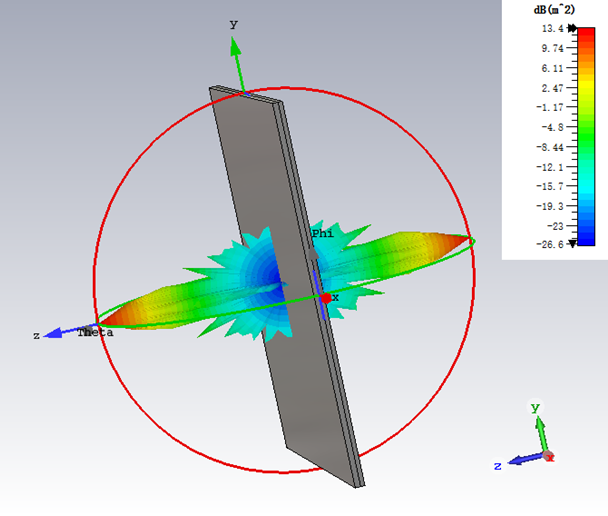


**Fig. S14** 3D radar wave scattering signal map of PEC


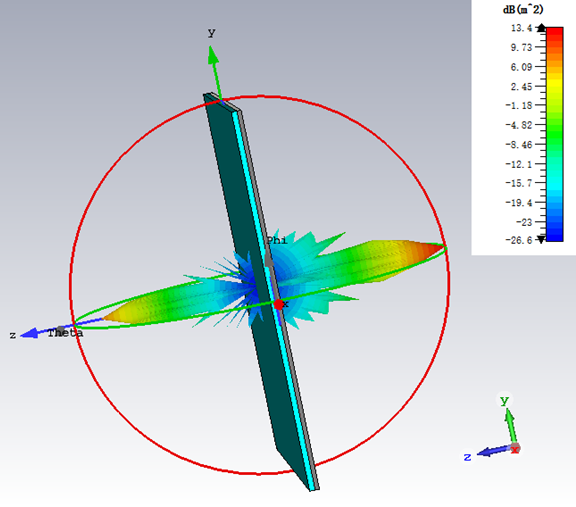


**Fig. S15** 3D radar wave scattering signal map of WCC-4

**Table S1** The comparison in RL_min_ and EAB_max_ with those reported carbides/carbon composites

| Absorbers | EAB_max_ | RL_min_ | References |
| --- | --- | --- | --- |
| Mo_2_C/C | 4.3 GHz (1.9mm) | -49.0 dB (2.6mm) | [68] |
| WM@C^a^ | 5.5 GHz (1.8mm) | -26.8 dB (2.7mm) | [69] |
| WC@CN | 5.1 GHz (1.6mm) | -42.3 dB (4.0mm) | [20] |
| WC/C | 4.9 GHz (1.5mm) | -55.0 dB (1.3mm) | [21] |
| SiC@C | 2.9 GHz (1.0 mm) | -27.6 dB (2.9mm) | [70] |
| Mxene@RGO | 4.7 GHz (2.0 mm) | -27.5 dB (4.0mm) | [71] |
| WCC-2 | 6.3 GHz (2.3 mm) | -72.0 dB (2.6mm) | This work |

^a^:WC/Mo_2_C@C
